# Diversification of a single ancestral gene into a successful toxin superfamily in highly venomous Australian funnel-web spiders

**DOI:** 10.1186/1471-2164-15-177

**Published:** 2014-03-05

**Authors:** Sandy S Pineda, Brianna L Sollod, David Wilson, Aaron Darling, Kartik Sunagar, Eivind A B Undheim, Laurence Kely, Agostinho Antunes, Bryan G Fry, Glenn F King

**Affiliations:** 1 Institute for Molecular Bioscience, The University of Queensland, 306 Carmody Road, St Lucia, QLD 4072, Australia; 2 Department of Molecular, Microbial & Structural Biology, School of Medicine, University of Connecticut, Farmington, CT 06030, USA; 3 Xenome, P.O. Box 1024, Indooroopilly Centre, QLD 4068, Australia; 4 CIMAR/CIIMAR, Centro Interdisciplinar de Investigação Marinha e Ambiental, Universidade do Porto, Rua dos Bragas, 177, 4050-123 Porto, Portugal; 5 Departamento de Biologia, Faculdade de Ciências, Universidade do Porto, Rua do Campo Alegre, 4169-007 Porto, Portugal; 6 Venom Evolution Lab, School of Biological Sciences, The University of Queensland, St Lucia, QLD 4072, Australia; 7 Current address: Monsanto Company, 800 N. Lindbergh Blvd, St. Louis, MO 63167, USA; 8 Current address: Faculty of Medicine, Health and Molecular Science-Queensland, Tropical Health Alliance, James Cook University, Cairns, Queensland, Australia; 9 Current address: The i3 Institute, University of Technology Sydney, Ultimo, NSW 2007, Australia

**Keywords:** Spider toxin, Spider venom, Hexatoxin, ω-hexatoxin, κ-hexatoxin, Australian funnel-web spider, Molecular evolution, Gene duplication, Positive selection, Negative selection

## Abstract

**Background:**

Spiders have evolved pharmacologically complex venoms that serve to rapidly subdue prey and deter predators. The major toxic factors in most spider venoms are small, disulfide-rich peptides. While there is abundant evidence that snake venoms evolved by recruitment of genes encoding normal body proteins followed by extensive gene duplication accompanied by explosive structural and functional diversification, the evolutionary trajectory of spider-venom peptides is less clear.

**Results:**

Here we present evidence of a spider-toxin superfamily encoding a high degree of sequence and functional diversity that has evolved via accelerated duplication and diversification of a single ancestral gene. The peptides within this toxin superfamily are translated as prepropeptides that are posttranslationally processed to yield the mature toxin. The N-terminal signal sequence, as well as the protease recognition site at the junction of the propeptide and mature toxin are conserved, whereas the remainder of the propeptide and mature toxin sequences are variable. All toxin transcripts within this superfamily exhibit a striking cysteine codon bias. We show that different pharmacological classes of toxins within this peptide superfamily evolved under different evolutionary selection pressures.

**Conclusions:**

Overall, this study reinforces the hypothesis that spiders use a combinatorial peptide library strategy to evolve a complex cocktail of peptide toxins that target neuronal receptors and ion channels in prey and predators. We show that the ω-hexatoxins that target insect voltage-gated calcium channels evolved under the influence of positive Darwinian selection in an episodic fashion, whereas the κ-hexatoxins that target insect calcium-activated potassium channels appear to be under negative selection. A majority of the diversifying sites in the ω-hexatoxins are concentrated on the molecular surface of the toxins, thereby facilitating neofunctionalisation leading to new toxin pharmacology.

## Background

Venoms have proven to be key evolutionary innovations for many divergent animal lineages [1,2]. Although the most extensively studied venoms are from the medically important scorpions, snakes, and spiders, venom systems are present in many other lineages including cnidarians, echinoderms, molluscs, fish, lizards, and mammals [1,2]. These venoms have evolved to serve a variety of purposes, including prey capture, competitor deterrence, and defense against predators. There has been considerable innovation both in the chemical composition of these venoms as well as the method of venom delivery, which includes barbs, beaks, fangs, harpoons, nematocysts, pinchers, proboscises, spines, spurs, and stingers [1,2].

From a molecular evolutionary perspective, the venoms of snakes are the best understood. There is now abundant evidence that snake venoms evolved by recruitment of genes encoding normal body proteins followed by extensive duplication, neofunctionalization, and in some instances relegation to the status of pseudogene [1,3-5]. In many cases, these genes have been explosively replicated to produce large multigene families. This process is analogous to the birth-and-death model of evolution proposed for multigene families involved in adaptive immunity, such as the major histocompatibility complex and immunoglobulin *V*_H_ genes [6]. However, the evolutionary trajectory is less clear for the venoms of spiders, scorpions, and molluscs, which are dominated by disulfide-rich peptides of mass 2–9 kDa [7-12]. These peptides typically possess high affinity and often-exquisite specificity for particular classes of ion channels and other nervous system targets [13-15]. These neurotoxic functions are perhaps not surprising given that the primary role of these venoms is to paralyse or kill envenomated prey [11,16,17].

**Figure 1:**
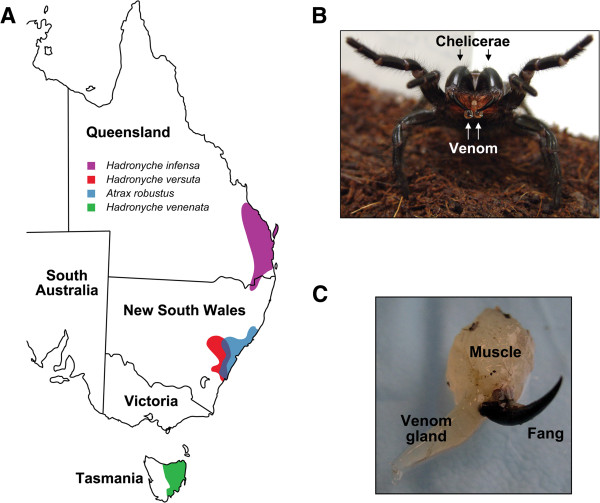
**Distribution, venom collection and venom-gland dissection of Australian funnel-web spider species used in this study. (A)** Map of the eastern half of Australia showing the distribution of the five species of Australian funnel-web spider used in this study. **(B)** Female funnel-web spider (*Hadronyche infensa*) from Fraser Island, QLD. In response to provocation, the spider has adopted a typical aggressive/defensive posture, with front legs and pedipalps raised and the fangs in an elevated position ready to strike. Note the drop of venom on each of the fang tips. **(C)** A single *H. versuta* venom gland that has been dissected from the surrounding muscle tissue. The venom gland in these and other mygalomorph spiders is located directly below the dorsal surface of the chelicerae.

In this study, we analysed toxin-encoding transcripts from five species of Australian funnel-web spider (Aranae: Mygalomorphae: Hexathelidae: Atracinae) from the genera *Atrax* and *Hadronyche*, representing a geographic spread of more than 2000 km (Figure 1), in order to provide insight into the evolutionary trajectory of the ω-hexatoxin-1 (ω-HXTX-1) family. ω-Hexatoxins (formerly known as ω-atracotoxins) are peptides comprising ~37 residues that were first isolated from the venom of the lethal Blue Mountains funnel-web spider *Hadronyche versuta*[18]. The ω-hexatoxins are major components in the venom of Australian funnel-web spiders [18-20] and they contribute significantly to prey immobilization by virtue of their ability to specifically block insect, but not vertebrate, voltage-gated calcium (Ca_V_) channels [17,18,20-22]. Their potent insecticidal activity has engendered interest in these peptides as bioinsecticides [11,17,23]. Proteomic analysis of *H. versuta* venom revealed a number of ω-HXTX-Hv1a paralogs [19], suggesting that this peptide toxin might belong to a multigene family. However, because the venom used in this previous study was pooled from several spiders, it was unclear whether these apparent paralogs are simply polymorphisms resulting from allelic variation. By using cDNA libraries obtained from a *single* spider, we demonstrate here that ω-HXTX-Hv1a is indeed part of a large multigene family that appears to have arisen from explosive gene duplication followed by extensive sequence divergence and neofunctionalization. Within this *superfamily* of toxins, we show that pharmacologically distinct toxin classes are evolving under starkly different selection pressures, with some toxin classes accumulating variation under episodic bursts of adaptation, while others remain constrained by negative selection. This work reinforces the idea that the chemical and pharmacological diversity present in spider venoms may have evolved from a relatively small number of ancestral genes.

## Results and discussion

### The ω-hexatoxins are expressed as prepropeptide precursors

**Figure 2:**
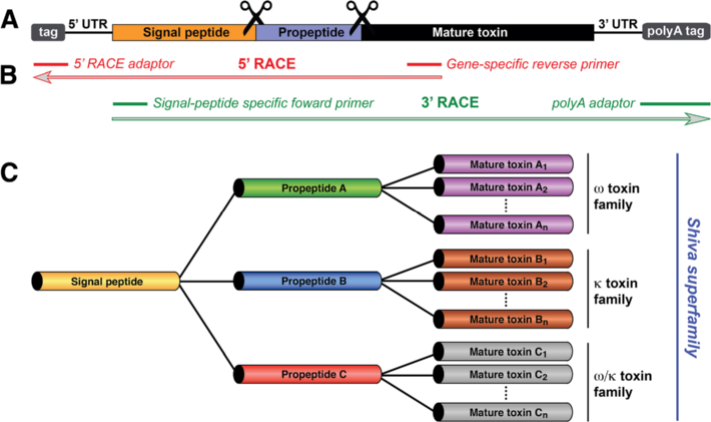
**Schematic representation of toxin precursors, overall RACE amplification strategy and identification of toxin superfamilies. (A)** Schematic representation of a typical spider peptide precursor showing the signal peptide in orange, the propeptide in purple, and the mature toxin in black. After translation, the signal and propeptide regions are proteolytically removed to yield a functional mature toxin. **(B)** General overview of the RACE protocol for sequencing hexatoxin transcripts. Adaptors are added to the 5’ and 3’ end of transcripts during cDNA library preparation. In both 3’ and 5’ RACE, gene-specific primers are used in the forward (3’ RACE) or reverse (5’ RACE) orientation to amplify full-length sequences. The resulting PCR products are then cloned and sequenced. **(C)** Schematic representation of the Shiva superfamily highlighting the combinatorial nature of spider-venom peptides.

**Figure 3:**
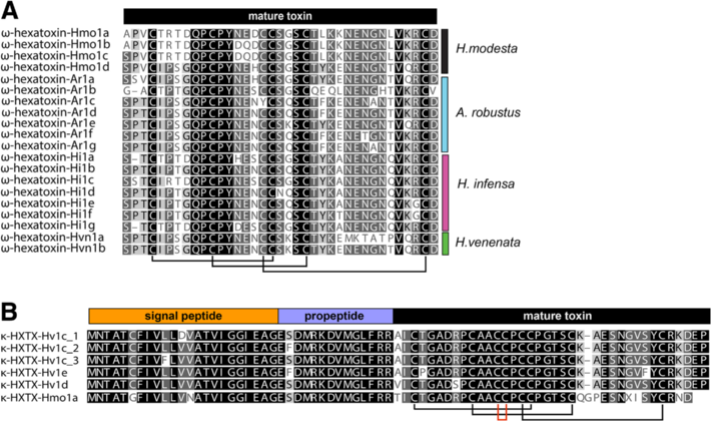
**Sequence alignments of ω and κ -hexatoxins. (A)** Sequence alignment of ω-HXTX-Hv1a paralogs from each species: *Hadronyche modesta* (Hmo1a–Hmo1d), *Atrax robustus* (Ar1a–Ar1g), *Hadronyche infensa* (Hi1a–Hi1g), and *Hadronyche venenata* (Hvn1a and Hvn1b). The level of residue conservation is graded from black (fully conserved across all paralogs) to dark grey (conserved in most toxins) to light grey (conserved in a majority of orthologs). The lines below the sequence alignment indicate the disulfide-bond connectivity of ω-HXTX-Hv1a. **(B)** Alignment of κ-HXTX-Hv1a paralogs from *Hadronyche versuta* and one ortholog from *Hadronyche modesta*. The level of residue conservation is graded as in panel (A) and the signal peptide, propeptide, and mature toxin regions are highlighted. The lines below the sequence alignment indicate the disulfide-bond connectivity of κ-HXTX-Hv1c, with the vicinal disulfide bond highlighted in red. Note that ω-HXTX-Ar1a (UniProt PF06357), ω-HXTX-Hi1a (UniProt P0C2L5), ω-HXTX-Hi1b (UniProt P0C2L6), ω-HXTX-Hi1c (UniProt P0C2L7), and κ-HXTX-Hv1c (UniProt P82228) have been previously isolated directly from venom.

RACE analysis was used to amplify transcripts encoding orthologs of ω-HXTX-Hv1a from four species of Australian funnel-web spider: *Atrax robustus*, *H. infensa*, *H. venenata*, and *H. versuta* (Figure 2). Multiple ω-HXTX-Hv1a orthologs were identified in each species (i.e., 24 paralogs encoding seven distinct mature toxins were identified in *H. infensa*, 18 paralogs encoding six mature toxins were identified in the Sydney funnel-web spider *A. robustus*, and eight paralogs encoding two mature toxins identified in the Tasmanian funnel-web spider *H. venenata*) (Figure 3A). A further eight paralogs encoding four distinct mature toxins were identified in the venom-gland transcriptome of *H. modesta* (Figure 3A). Thus, the amino acid sequence diversity previously reported for ω-HXTX-1 based on analysis of pooled venom samples [19] is due to expression of multiple related transcripts in a single spider rather than allelic variation. The almost complete conservation of the signal sequence, as well as the pattern of conserved cysteines in the mature toxin (Figure 3A), indicates that these ω-HXTX-Hv1a homologs arose by duplication and sequence divergence of the original toxin-encoding gene.

All of the ω-HXTXs are expressed as prepropeptide precursors that are posttranslationally processed to yield the mature toxin sequence (Figure 2A). The highly hydrophobic 22-residue signal sequence is of similar length to that reported for peptide-toxin precursors from spiders, scorpions, and cone snails [8]. The 20-residue propeptide sequence is highly acidic, with a net charge of -4, a feature that has been noted for numerous spider-toxin propeptide sequences [24-28] but which is not characteristic of toxin precursors from other venomous animals. Moreover, the presence of a propeptide region contrasts with most scorpion-toxin precursors in which the signal sequence is fused directly to the mature toxin without an intervening propeptide [8]. The reason for the highly acidic propeptide region in spider toxin precursors remains to be determined, but it may be related to specific interactions between the toxin precursor and components of the secretory and/or protein folding pathway in spider venom glands.

The propeptide sequence terminates with a dibasic Arg-Arg signature; dibasic sequences are common recognition sites for proteolytic removal of propeptide segments in neuropeptide precursors from both vertebrates [29] and invertebrates [30]. While Arg is the terminal residue in virtually all known spider-toxin propeptide sequences, the penultimate residue is variable, though it is commonly Asp, Glu, or Lys [25,26,31-33].

### ω-hexatoxins belong to a large toxin-gene superfamily

Orthologs of ω-HXTX-Hv1a were identified in all five species of Australian funnel-web spider examined in this study. These species are distributed along the eastern seaboard of Australia with a geographic spread of more than 2000 km (Figure 1). The hexathelids are a group of approximately 40 species divided into three genera: *Atrax*, *Hadronyche* and *Illawarra*[34-36]. They are adapted to forest environments but can also be found in habitats that range from montane herblands and open woodland to closed forest [36]. Conservation of the ω-hexatoxin family of toxins over this wide range of environments and differing prey distributions implies that there has been strong evolutionary pressure to maintain these peptides as part of the venom arsenal, which is perhaps not surprising given that they are broadly active against many different arthropods [11,17,37].

In addition to obvious homologs of ω-HXTX-Hv1a, the RACE and transcriptomic analyses revealed additional families of toxins that had almost identical signal sequences to the ω-HXTX-1 transcripts, but divergent propeptide and mature toxin sequences. We named one of these families the ω/κ-HXTX family (described as U-ACTX in [38]). The ω/κ-HXTX peptides appear to be distributed in two of the species examined (*A. robustus* and *H. versuta*); this reinforces the idea that these toxins most likely arose ancestrally by duplication of a ω-HXTX-1 gene followed by hypermutation of the propeptide and mature-toxin regions in order to create a new function (neofunctionalization). The conservation and radiation of these toxins across this family of spiders implies that they are not nonfunctional relics of an explosive radiation of this toxin-gene superfamily, and we confirmed this by showing that recombinant ω/κ-HXTX-Hv1a is highly insecticidal [38]. The high insecticidal potency of this family of peptides is believed to result from a synergistic effect on insect voltage-gated calcium (Ca_V_) channels and calcium-activated potassium (K_Ca_) channels [38].

RACE analysis of the venom-gland cDNA library from *H. versuta* also led to amplification of transcripts encoding the insecticidal toxin κ-HXTX-Hv1c [39], and sequencing of the venom-gland transcriptome from *H. modesta* also uncovered an ortholog of this toxin (Figure 3B). This was entirely unexpected since this toxin has a vastly different primary structure to ω-HXTX-Hv1a [39]. Moreover, in addition to the six conserved cysteine residues in ω-HXTX-Hv1a that form an inhibitor cystine knot (ICK) motif [40,41], κ-HXTX-Hv1c contains two additional cysteine residues that form an extremely rare vicinal disulfide bond [42-44]. Furthermore, in contrast to ω-HXTX-Hv1a, which blocks insect Ca_V_ channels, κ-HXTX-Hv1c is a potent and specific blocker of K_Ca_ channels [45]. Nevertheless, the near identity of the signal sequence in these two toxin families and the conservation of cysteine residues in the mature toxin indicate that they evolved from the same ancestral toxin gene and are members of the same gene superfamily.

We did not find orthologs of κ-HXTX-Hv1c in any of the other three species of Australian funnel-web spider (*H. infensa, A. robustus, and H. venenata*). However, κ-HXTX-Hv1a, κ-HXTX-Hv1b, and κ-HXTX-Hv1c are expressed at very low levels in *H. versuta* venom [42], and consequently we cannot rule out the possibility that these toxins are present in the venom of the other three spiders but the transcript levels are too low to be detected using the methods employed here.

### The Shiva superfamily of peptide toxins

It has previously been suggested that superfamilies of spider-venom peptides evolved from a single ancestral gene via explosive gene duplication [8]; the work described here further supports this idea as it is clear that the ω-HXTXs, ω/κ-HXTXs, and κ-HXTXs belong to a large superfamily of toxins that arose via gene duplication (Figure 2C). We have chosen to name spider-toxin gene superfamilies after deities of death and destruction since the major biological role of these toxins is to paralyze and/or kill envenomated prey. Accordingly, we have named the ω/κ-HXTX/ω-HXTX/κ-HXTX gene superfamily after the Hindu deity Shiva, commonly known as the “destroyer”.

**Figure 4:**
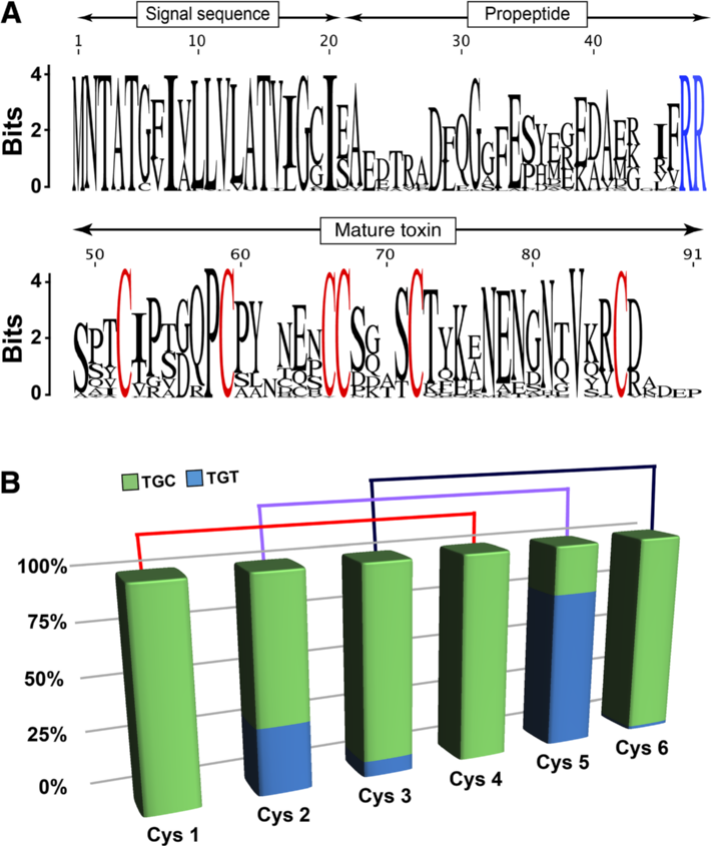
**Sequence logo and codon usage analysis from the Shiva superfamily. (A)** Sequence logo [54] based on alignment of prepropeptides from the Shiva superfamily. There is a much higher level of sequence conservation within the signal peptide than within the propeptide and mature toxin regions. Note, however, that the cysteine residues that form the cystine-knot motif in the mature toxin and the Arg-Arg protease recognition site that terminates the propeptide region are both completely conserved (highlighted in blue and red, respectively). **(B)** Codon usage for the six-cysteine residues that form the cystine-knot motif. Note the strong bias for TGC at cysteine positions 1, 3, 4, and 6. Shown above the histogram is the disulfide bridge arrangement for the six cysteines as inferred from the 3D structures of ω-HXTX-Hv1a and κ-HXTX-Hv1c.

Sequence logos were previously used to analyse differences in the level of sequence conservation between the three parts of the ω-HXTX toxin precursor, namely the signal peptide, propeptide, and mature toxin [8]. A revised logo analysis of the Shiva superfamily (Figure 4A) that incorporated all of the new sequences and species reported here reinforced the dichotomy in evolutionary forces affecting various elements of the toxin precursor. The signal peptide has clearly been highly conserved throughout the evolution of this toxin superfamily and it is presumably under negative selection in order to ensure that these toxins are directed to the appropriate secretory pathway. In contrast, there is significant sequence variation in both the propeptide and mature toxin sequences, with two notable exceptions. First, in contrast to the highly variable upstream region of the propeptide sequence, the C-terminal proteolytic recognition signal (Arg-Arg) is completely preserved (Figure 4A). Presumably there has been strong selection pressure to ensure processing of the propeptide by a specific protease. Second, in contrast to the overall low level of conservation of the mature toxin sequence, the cysteine residues, which direct the three-dimensional (3D) fold of the toxins, are completely conserved (Figure 4A). The marked variation in levels of sequence conservation between the spider-toxin signal sequence and the propeptide and mature toxin regions is reminiscent of that observed for superfamilies of cone snail toxins [46-51].

There are two striking differences between the Shiva superfamily precursors and transcripts encoding human neuropeptides and other secreted proteins. First, whereas precursors of human neuropeptides often encode multiple mature neuropeptide sequences [29,52], we and others have not found any examples of spider-toxin transcripts that encode more than a single mature toxin sequence. Secondly, in direct contrast to the toxin precursors, the sequence of the mature human neuropeptide(s) is usually strongly conserved whereas there is significantly more variability in the signal sequence. This is perhaps not surprising given that human neuropeptides usually act on a single well-defined molecular target whereas spider toxins typically target a specific subtype of receptor or ion channel that nevertheless might vary significantly in primary structure between prey taxa (Note that most spiders are generalist predators that target a phylogenetically diverse range of prey). Thus, expressing a family of related toxins in the venom (essentially a mini-combinatorial peptide library) might ensure that the desired receptor/ion channel is targeted, regardless of prey taxa.

### Position-specific cysteine codon bias

Mature ω-HXTXs contain three disulfide bonds with 1–4, 2–5, 3–6 connectivity. These disulfides form an ICK motif that provides these toxins with a high degree of chemical, thermal and biological stability [53]. Although it is clear from a protein structure viewpoint why these six cysteine residues need to be strictly maintained in order to preserve the toxin’s 3D scaffold, one would not expect to find a preference for either one of the two possible cysteine codons (TGT and TGC). Intriguingly, however, previous analysis of ω-HXTX precursors revealed a strong bias for TGC at four of the six cysteine positions in the mature toxin region [8]. An extended logo analysis [54] incorporating all of the newly discovered sequences reported in this study corroborated the previously observed codon bias (Figure 4B). We found an extreme TGC codon bias for the four cysteine residues that form the 1–4 and 3–6 disulfide bridges in the ω-HXTX family but not for the two cysteines that form the 2–5 disulfide bond (Figure 4B). The observed position-specific codon bias is not simply a manifestation of global codon bias in these spiders as we have observed a preference for TGT as opposed to TGC for cysteine residues in other hexatoxin superfamilies (data not shown). Moreover, we did not observe extreme codon bias for any other conserved residue in the mature hexatoxins.

Position-specific cysteine-codon bias has also been observed in superfamilies of cone snail toxins and it has been proposed that these codons might serve as attractants for a mutator complex that includes a poorly processive and highly mutagenic polymerase (e.g., DNA Pol V) that promotes radiation of the toxin superfamily by facilitating hypermutation of the mature toxin region [49,50]. However, there is currently no direct evidence that cysteine-codon bias plays a part in directing the evolution of spider or cone snail toxins.

### Molecular evolution analyses

We utilized various state-of-art molecular evolutionary assessment methods to determine the influence of natural selection on the evolution of genes encoding Shiva superfamily toxins (see *Methods* section for full details of the selection analyses). The one-ratio model, the simplest of the codon-specific models, estimated the non-synonymous-to-synonymous nucleotide-substitution rate ratio (ω) to be 0.64, 1.06 and 0.69 for the ω-HXTXs, κ-HXTXs, and combined Shiva superfamily dataset, respectively (Additional file 1: Table S1–3). This highly conservative model can only detect positive selection when ω, averaged over all sites along the lineages in a phylogenetic tree, is significantly greater than one. As lineage-specific models of PAML, such as the one-ratio model, often fail to detect positive-Darwinian selection that only affects certain sites in proteins, we also employed site-specific models (Table 1: codon numbers based on κ-HXTX-Hv1c_2 and ω-HXTX-Ar1a_1; Additional file 1: Table S1–3). Model 8 estimated ω of 0.69, 1.06 and 0.78 for the ω-HXTXs, κ-HXTXs, and the combined Shiva superfamily dataset, respectively (Table 2 and Additional file 1: Table S1–3). Although the computed ω for the κ-HXTXs was >1, the assessment was not statistically significant (p > 0.05) in comparison with the null model (M7 β). The Bayes Empirical Bayes (BEB) approach implemented in M8 was only able to identify one positively selected site in the combined toxin dataset (Table 2 and Additional file 1: Table S3). Thus, the site-specific models failed to detect the influence of adaptive selection pressures in shaping evolution of the Shiva superfamily. In contrast, the more advanced Fast, Unconstrained Bayesian AppRoximation (FUBAR) [55,56] implemented in HyPhy detected a handful of positively selected sites in both the ω-HXTXs and the combined dataset (Table 1).

**Table 1:** Nucleotide and complementary protein analyses for ω toxins

**Site**^**a**^		**CodeML**		**Tree SAAP**		**Accessible surface area**^**f**^
**Codon**	**Amino Acid**	**M2a**^**b**^	**M8**^**c**^	**Property**^**d**^	**Magnitude**^**e**^	
**21**	**E**	0.99 ± 0.38	0.87 ± 0.42	**-**	**-**	**-**
		(0.201)	(0.257)			
**40**	**V**	1.41 ± 0.49	1.47 ± 0.37	*M*_*W*_*, M*_*V*_*, V*^*0*^*,* μ	8, 8, 8, 8	42.0
		(0.589)	(0.842)			Partially exposed
**44**	**S**	1.25 ± 0.37	1.28 ± 0.41	*M*^*W*^*, M*_*V*_*, V*^*0*^*,* μ	8, 8, 8, 8	82.1
		(0.420)	(0.647)			Exposed
**53**	**H**	1.62 ± 0.63	1.55 ± 0.36	**-**	**-**	0.0
		(0.749)	(0.929)			Buried
**57**	**G**	1.39 ± 0.48	1.45 ± 0.38	**-**	**-**	57.3
		(0.570)	(0.825)			Exposed
**60**	**T**	0.80 ± 0.44	0.71 ± 0.41	**-**	**-**	49.7
		(0.122)	(0.154)			Exposed
**64**	**N**	*0.53 ± 0.40*	*0.51 ± 0.30*	**-**	**-**	100.0
		(0.031)	(0.036)			Exposed
**69**	**T**	1.34 ± 0.45	1.38 ± 0.40	**-**	**-**	59.8
		(0.507)	(0.751)			Exposed
**72**	**R**	1.10 ± 0.33	1.01 ± 0.42	**-**	**-**	0.0
		(0.26)	(0.374)			Buried

^a^Sites detected as positively selected using the integrative approach.

^b^M2a Bayes empirical Bayes (BEB) posterior probability and post-mean ω indicated in parentheses.

^c^M8 Bayes empirical Bayes (BEB) posterior probability and post-mean ω indicated in parentheses.

^d^Amino acid property under selection (*M*_*W*_: molecular weight; *M*_*V*_*:* molecular volume; *V*^*0*^*:* partial specific volume; *μ:* Refractive index).

^e^Magnitude if selection on the amino acid property.

^f^Accessible surface area of 10–20% corresponds to buried residues, 40–50% indicates partially exposed amino acid residues, and ≥50% indicates solvent exposed residues.

**Table 2:** Molecular evolution of ω and κ toxins from Australian funnel-web spiders

	**FUBAR**^**a**^	**MEME**^**b**^	**PAML**^**c**^	
			**M8**	**M2a**
ω	ω >1^d^: 3	7	0	0
toxins	ω <1^e^: 5		0	0
			0.69	0.73
κ	ω >1^a^: 1	0	0	0
toxins	ω <1^b^: 0		0	0
			1.06^NS^	1.06^NS^
ALL	ω >1^a^: 3	8	1	0
toxins	ω <1^b^: 7		(0 + 1)	0
			0.78	0.83

^a^Fast, Unconstrained BayesianApproximation (FUBAR).

^b^Sites detected as experiencing episodic diversifying selection (0.05 significance) by mixed effects model evolution (MEME).

^c^Positively selected sites detected using the Bayes empirical approach implemented in the site models M8 and M2a. Number of positively selected sites detected at the posterior probability ≥0.99 and 0.95 are indicated in parenthesis. ω computed using M8 and M2a are also presented.

^d^Number of sites evolving under the influence of pervasive diversifying selection, detected by FUBAR at 0.9 posterior probability.

^e^ Number of sites evolving under the influence of pervasive purifying selection, detected by FUBAR at 0.9 posterior probability.

**ω =** mean dN/dS.

**NS =** not significant at 0.05 compared to the null model (M7: beta).

**Figure 5:**
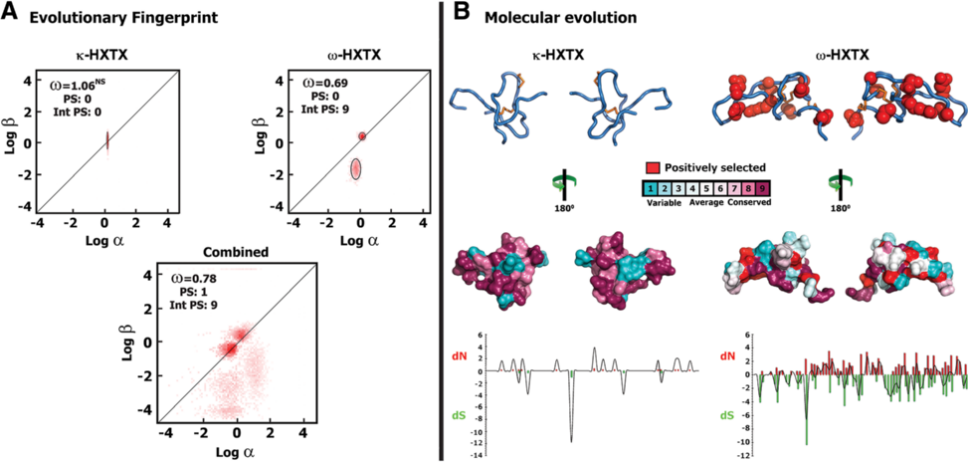
**Molecular evolution analyses of κ- and ω-HXTXs. (A)** Evolutionary fingerprint of κ- and ω-HXTXs. Estimates of the distribution of synonymous (α) and non-synonymous (β) substitution rates inferred for the κ-HXTXs, ω-HXTXs, and the combined Shiva superfamily dataset. The ellipses reflect a Gaussian-approximated variance in each individual rate estimate, and colored pixels show the density of the posterior sample of the distribution for a given rate. The diagonal line represents the idealized neutral evolution regime (ω = 1), while points above and below the line correspond to positive selection (ω > 1) and negative selection (ω < 1), respectively. The ω for site model 8, along with the total number of positively selected sites detected by its Bayes Empirical Bayes (BEB) approach and the number of episodically diversifying sites detected by the mixed effects model of evolution (MEME), are also indicated. **(B)** Molecular evolution of Shiva superfamily toxins from Australian funnel-web spiders. 3D homology models are shown with their molecular surface colored according to the evolutionary conservation of amino acids (see color key); the location of positively selected sites is shown in red in space-fill models and as red spheres in wireframe models. A line plot is also provided to highlight the relative accumulation of dN versus dS, estimated using the M0 model of PAML. NS: Not significant.

Site-specific models for detecting positive selection work best when detecting pervasive selection pressures. However, the majority of positively selected sites are often subjected to transient or episodic adaptations. When the majority of lineages evolve under the influence of negative selection, they mask the signal of positive selection that influences only a small number of lineages. In such scenarios, the aforementioned analyses may fail to detect the influence of positive selection. To address the shortcomings of the aforementioned approaches, we employed the advanced Mixed Effects Model Evolution (MEME) [57], which uses fixed effects likelihood (FEL) along the sites and random effects likelihood (REL) across the branches to detect episodic diversifying selection. MEME is capable of identifying both pervasive and episodic adaptations. MEME identified 7 and 8 episodically diversifying sites in the ω-HXTXs and combined toxin dataset, respectively (Table 2), highlighting the vital role of episodic diversifying selection in shaping the evolution of these spider toxins. Six out of eight episodically diversifying sites (75%) were located on the molecular surface of the toxins (Table 1 and Figure 5B) with their side chains completely or partially exposed to solvent, suggesting that they could act as pharmacological sites and participate in prey envenomation; these findings are also in agreement with the selection forces found on the surface of the SGTx toxin family from the venom of the African Baboon spider *Scodra grisiepies*[58]. Rapid Accumulation of Variations in Exposed Residues (RAVER), where the toxin molecular chemistry undergoes hypervariations under the influence of positive Darwinian selection and focal mutagenesis [59], has been documented in a plethora of venom-components from a wide diversity of venomous animal lineages [59-64]. Since the synthesis and secretion of venom proteins is energetically expensive [65-67], mutations that disrupt the structure/function of proteins are filtered out of the population by negative selection over time, favoring the conservation of catalytic and structurally important residues. RAVER not only aids in generation of a rapidly variable toxin molecular surface biochemistry, but it also ensures the conservation of structurally and functionally important residues. Accumulation of variations on the molecular surface of the toxin is advantageous as the altered surface chemistry might lead to new toxin functions (neofunctionalisation).

To derive further support for the positively selected sites detected by nucleotide analyses, we employed a complementary protein-level approach implemented in TreeSAAP (Table 1). TreeSAAP identified two positively selected sites in the ω-HXTXs that were in common with the sites identified by site-model 8 of PAML (Table 1). Evolutionary fingerprint analyses (Figure 5A) clearly revealed several residues in the ω-HXTXs and the combined toxin dataset that evolve under the influence of positive selection, while a majority of residues in the κ-HXTXs remained under evolutionary constraint (Figure 5A,B). Thus, evolution of the ω-HXTXs has been significantly influenced by short bursts of episodic adaptations, while the κ-HXTXs appear to be under negative selection.

**Figure 6:**
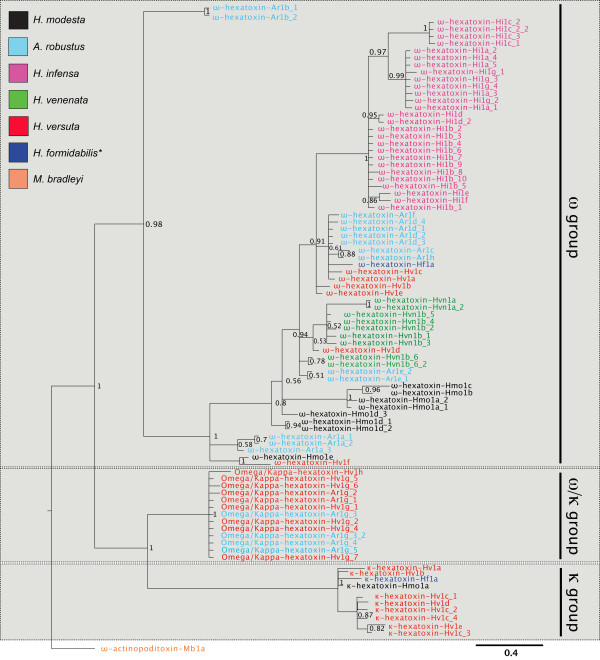
**Bayesian phylogenetic tree representing the molecular evolutionary history of the Shiva superfamily toxins.** The tree shows the split between the three main toxin classes (ω, κ, and ω/κ). ω-Actinopoditoxin-Mb1a from the Eastern mouse spider *Missulena bradleyi* was used as the outgroup. Toxins belonging to each species are highlighted in the following colours: *H. versuta*, red; *H. modesta*, black; *H. venenata*, green; *H. infensa*, magenta; *A. robustus*, pale blue; *H. formidabilis*, dark blue; ω-actinopoditoxin-Mb1a from *M. bradleyi*, orange. *denotes a species not sequenced as part of this study; the sequence was downloaded from UniProt under accession number P83588.

Phylogenetic analysis revealed that the κ-HXTXs form a separate clade to the ω-HXTXs, rendering the Shiva superfamily non-monophyletic (Figure 6). There are also significant variations within the ω-HXTXs suggestive of functional diversification (Figure 6). The “hybrid” ω/κ-HXTXs exhibit functional characteristics of both the ω-HXTXs and κ-HXTXs as they block Ca_V_ channels (like the ω-HXTXs) as well as K_Ca_ channels (like the κ-HXTXs). The functional activity of the ω/κ-HXTXs combined with their relative phylogenetic placement and cysteine pattern indicates that they are structurally and functionally intermediate between the ω- and κ-HXTXs. The evolution of new cysteine residues to create the vicinal disulfide bond in the κ-HXTXs potentiated toxin activity on K_Ca_ channels, since mutagenesis and analogue studies indicate that this vicinal disulfide bond is the most critical part of the K_Ca_ pharmacophore [43,45,68].

### Constraints on mutation of the mature toxin sequence

**Figure 7:**
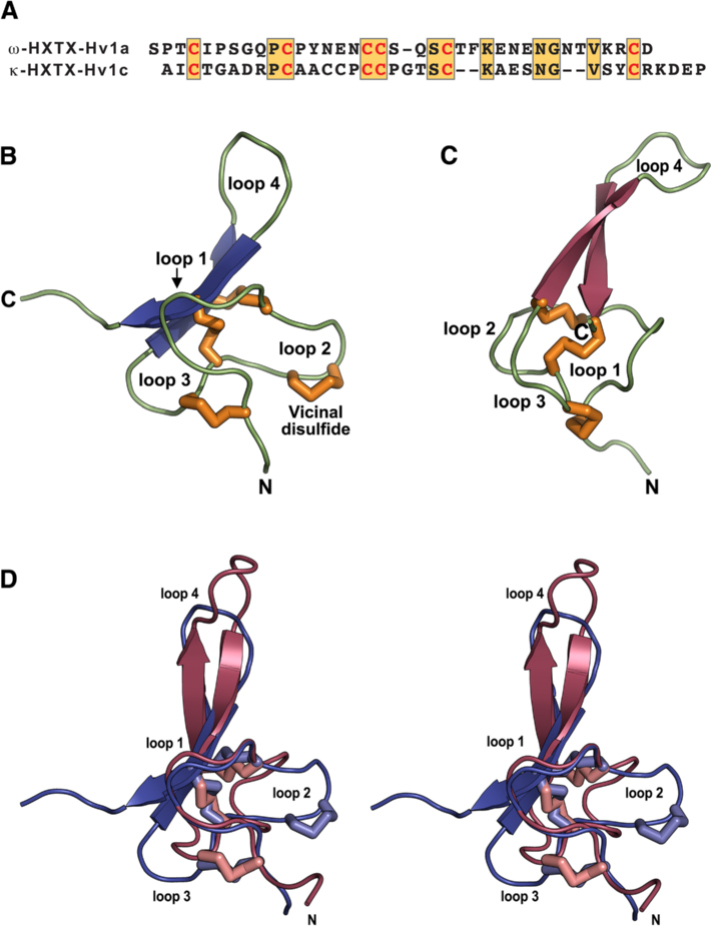
**Structural comparisons of κ-HXTX-Hv1c and ω-HXTX-Hv1a. (A)** Comparison of the primary structures of κ-HXTX-Hv1c and ω-HXTX-Hv1a. Identities are boxed and shaded orange while the conserved cysteines that form the cystine-knot motif in each toxin are coloured red. **(B)** Richardson representation of the 3D structure of κ-HXTX-Hv1c (PDB accession code 1DL0; [42]) and **(C)** ω-HXTX-Hv1a (PDB accession code 1AXH; [18]). Disulfide bonds are shown as orange tubes. The inter-cystine loops and the N- and C-termini are labelled. **(D)** Stereo-view of an overlay of the 3D structures of κ-HXTX-Hv1c (dark blue with light-blue disulfide bonds) and ω-HXTX-Hv1a (raspberry with light-salmon disulfide bonds). The structural alignment, which was automatically generated by DaliLite [70], yielded a root mean square deviation of 2.4 Å over the backbone atoms of the 28 aligned residues (Ala1–Ala11, Pro15–Pro18, Ser21–Asn27, and Gly28–Arg33 in κ-HXTX-Hv1c versus Pro2–Pro12, Asn16–Ser19, Ser21–Asn27, and Thr32–Asp37). The inter-cystine loops and the N-terminus are labelled. Figures generated using MacPyMOL [91].

It is generally considered that conservation of the cysteine scaffold in toxin-gene superfamilies is critical for conserving the toxin’s 3D fold [35]. However, the incredible disparity in the amino acid sequence between ω-HXTX-Hv1a and κ-HXTX-Hv1c (Figure 7A) begs the question of whether this is reflected in a significant difference in their 3D structures, despite their common cystine-knot scaffold. The 3D structure of both toxins has been determined previously using homonuclear NMR spectroscopy [18,42] and their pharmacophores elucidated using alanine scanning mutagenesis [17,21,43,69].

Figure 7B and C show schematic representations of the 3D structure of κ-HXTX-Hv1c and ω-HXTX-Hv1a, respectively. The two toxins can be considered to comprise four inter-cystine loops, which are labelled 1–4 from N- to C-terminus. Although there is an obvious similarity in the disposition of the three centrally located disulfide bridges that form the cystine-knot motif in each toxin, the overall topology of the toxins, as well as the size and relative orientation of the four inter-cystine loops, appears quite different. However, the structural overlay in Figure 7D, which was generated automatically by the DaliLite structural alignment program [70], reveals that the two structures are in fact remarkably similar.

The DaliLite alignment yields a root mean square deviation of 2.4 Å over the backbone atoms of the 28 aligned residues, indicating that the two toxins are indeed structural homologs. The three central disulfide bridges and loop 1 align remarkably well. Loop 4, which encompasses the β-hairpin present in both toxins, also aligns well except for the four-residue insertion in ω-HXTX-Hv1a (Figure 7A), which increases the size of the hairpin loop at the tip of Loop 4. The major structural differences between the two toxins are the very different orientations of loops 2 and 3. However, these structural variations cannot disguise the fact that the two toxins essentially conform to the same 3D scaffold despite their extraordinary sequence divergence (16% identity if the cysteine framework is excluded). This ability to maintain a consistent molecular architecture despite massive variation in the inter-cystine loop sequences has important implications for the mechanism by which this superfamily of peptide toxins has evolved.

## Conclusions

Spiders and other venomous animals rely on the production of pharmacologically complex venoms for defense, prey capture, and competitor deterrence. The major components of most spider venoms are disulfide-rich peptides that have evolved to target a wide range of receptors and ion channels in the insect nervous system. The ω-HXTX and κ-HXTX families were the first peptides isolated from Australian funnel-web spiders that were shown to be insecticidal [17]. Analysis of all transcripts encoding these peptides showed that they are initially expressed as prepropeptides that are proteolytically processed to yield a 36–37 residue mature peptide that contains three disulfide bridges that form an ICK motif plus a non-canonical vicinal disulfide bond in the κ-HXTXs.

The extreme diversity of primary structure within the Shiva toxin superfamily suggests that there have been few evolutionary restraints on sequence diversification outside of the disulfide bridges that direct the 3D fold of these peptides. The ω-HXTXs, in particular, seem to have evolved under the influence of positive Darwinian selection in an episodic fashion, whereas the κ-HXTXs appear to be constrained by negative selection pressures. Functional assessments of these toxins should shed further light on why they have adopted quite contrasting molecular evolutionary regimes. ω-HXTXs were also found to have adopted RAVER, where a large number of the episodically diversifying sites are concentrated on the molecular surface, facilitating the generation of novel pharmacological sites. These toxins may therefore be good candidates for *in vitro* evolution studies designed to produce modified peptides with desired therapeutic [14] or agrochemical [11] properties. Most importantly, this study reinforces the idea that the remarkable chemical and pharmacological complexity of spider venoms may be derived from a relatively small number of ancestral genes.

## Methods

### Identification of ω-HXTX-1 homologs via rapid amplification of cDNA ends

Venom-gland cDNA libraries were prepared from *individual* specimens of the following species of Australian funnel-web spider (Arthropoda: Chelicerata: Arachnida: Araneae: Opisthothelae: Mygalomorphae: Hexathelidae): *Hadronyche infensa*, *H. versuta*, *H. venenata*, and *Atrax robustus*, which were collected from geographically distinct regions of Australia (Figure 1A). Spiders were cooled to -20°C for 40–60 min, then paired venom glands were carefully dissected from each specimen (Figure 1C). Each *pair* of venom glands was combined, then polyA + mRNA was extracted using a QuickPrep Micro mRNA Purification Kit (Amersham Pharmacia Biotech/GE Healthcare Life Sciences, Rydalmere, NSW, Australia) and stored at -20°C until further use. cDNA libraries were constructed using a Marathon cDNA Amplification Kit (Clontech Laboratories, Mount View, CA, USA). From the mRNA template, single-stranded cDNA was constructed using Superscript III reverse transcriptase (Life Technologies, Grand Island, NY, USA) and a poly(dT) anchor primer (Echoclonanch-2, GGGCAGGT_17_). Second-strand synthesis was performed according to the kit specifications except the cDNA was purified using a Concert Rapid PCR Purification kit (Gibco/Life Technologies) instead of a phenol/chloroform extraction. A Marathon cDNA Amplification adaptor (Clontech Laboratories) was then ligated to the double-stranded cDNA. After overnight ligation, the sample was precipitated using 10 μl of 5% w/w glycogen, 10 μl of 3 M sodium acetate pH 5.2, and 100 μl of 100% ethanol at -20°C. The sample was subsequently washed with 80% ethanol and dried for 10 min prior to resuspension in Tris-EDTA buffer.

Transcripts encoding ω-HXTX-Hv1a and paralogs/orthologs thereof were subsequently obtained via rapid amplification of cDNA ends (RACE) (Figure 2B) [71]. A redundant 3’ PCR primer based on residues 24–31 of ω-HXTX-Hv1a (ω-HV1 5’ RTTNCCRTTYTCRTTYTCYTTRAA 3’) was used in conjunction with a 5’ universal adaptor primer in a 5’ RACE experiment designed to extract information about the upstream region of the ω-HXTX-Hv1a transcript (EchoAP1: 5’ CACCCCTAATACGACTCACTATAGG 3’). A gene-specific primer for 3’ RACE was then designed based on the leader sequence obtained from the 5’ RACE experiment (3’ RACE primer: 5’ TGCTGCAATATGAATACCGC 3’. This primer was used in combination with the Echoclonanch-2 oligo(dT) primer (5’ GGGCAGGTTTTTTTTTTTTTTTTT 3’) to generate transcripts that encode a signal sequence homologous to that of ω-HXTX-Hv1a.

PCR products were extracted from agarose gels using a Gibco gel purification kit, precipitated using Pellet Paint Co-Precipitant kit (Novagen/EMD Millipore, Billerica, MA, USA), then phosphorylated with kinase in preparation for cloning. PCR products were then ligated into pSMART and transformed into *E. cloni* cells (Lucigen, Middleton, WI, USA) using the Lucigen CloneSmart Blunt Cloning kit. Transformed clones were cultured for one hour in Recovery Medium, then plated with 50 μg/mL ampicillin to allow for overnight growth. PCR screening was then used to select colonies with the expected insert size for DNA sequencing. DNA sequences (and the corresponding protein sequences) were collated and analysed using Geneious Pro, version 3.8.5 [72] and signal sequence cleavage sites were predicted using SignalP, version 3.0 [73].

### Identification of ω-HXTX-1 homologs via transcriptomics

Paired venom glands from *Hadronyche modesta* were dissected out and pooled. Total RNA was extracted using the standard TRIzol® Plus method (Invitrogen/Life Technologies) according to the manufacturer’s protocol. One microgram of Total RNA was used to construct a cDNA library using the CREATOR™ SMART™ cDNA library construction kit (Clontech Laboratories) following the manufacturer’s protocol. Briefly, total RNA was reversed transcribed using the SMART™ Moloney Murine Leukemia virus (MMLV) reverse transcriptase. Second-strand synthesis was completed using long distance polymerase chain reaction (PCR) as follows: 1 min at 95°C followed by 20 cycles of 1 min at 95°C and 6 min at 68°C. Products were then digested and size fractionated using a CHROMA SPIN-400 DEPC-H_2_O column (Clontech Laboratories) and then ligated into the pDNR-lib donor vector. Recombinant plasmids were electroporated into E-shot™ DHB10™-T1^R^ electro competent cells (Invitrogen/Life Technologies). 384 clones were randomly selected and sequenced by capillary electrophoresis on an Applied Biosystems 3730×l DNA analyzer (Applied Biosystems/Life Technologies) at the Brisbane node of the Australian Genome Research Facility (AGRF). Sequences were processed so vector and polyA + tails were clipped using CLC Main Work Bench (CLC-Bio), and the Blast2GO bioinformatic suite [74,75] was used to provide Gene Ontology, BLAST and domain/Interpro annotation. Signal sequence cleavage sites were predicted using SignalP, version 3.0 [73].

### Nomenclature

In accordance with the recently introduced systematic nomenclature for naming peptide toxins from venomous animals [76], ω-ACTX-Hv1a [18] and J-ACTX-Hv1c [39] have been renamed ω-HXTX-Hv1a and κ-HXTX-Hv1a, respectively, and the various paralogs and orthologs uncovered in this study have been named accordingly. Briefly, the Greek letter denotes the molecular target of the peptide, followed by the generic name indicating the family from which the toxin is derived; in this case the abbreviation is HXTX for hexatoxin. After the generic family name, a two-letter abbreviation is used to denote the genus and species, indicated by upper and lowercase letters respectively (i.e., Hv for *H. versuta*, Hi for *H. infensa,* etc.). The name of the species is immediately followed by a numeral that helps to distinguish different toxins with similar pharmacology and this number is followed by letter that denotes the paralog number (this is based on the number of different encoded mature toxin sequences).

### Molecular evolution analyses

A total of 73 nucleotide and 90 peptide sequences were aligned using the default settings in Geneious Pro, version 3.8.5 [72] then manually adjusted for optimal alignment prior to the following molecular evolution analyses (see Additional file 1: Figure S1 and S2).

#### Test for recombination

To overcome the effects of recombination on the phylogenetic and evolutionary interpretations [77], we employed Single Breakpoint algorithms implemented in the HyPhy package and assessed the effect of recombination on all the toxin forms examined in this study [78,79]. When potential breakpoints were detected using the small sample Akaike information criterion (AIC), the sequences were compartmentalized or partitioned before conducting selection analyses to allow the recombining units to have distinct phylogenies (as described in [80,81]).

#### Selection analyses

We evaluated the influence of natural selection on the toxins using maximum-likelihood models [82,83] implemented in CODEML of the PAML software [84]. We employed site-specific models that estimate positive selection statistically as an ω value significantly greater than 1. We compared likelihood values for three pairs of models with different assumed ω distributions as no *a priori* expectation exists for ω: M0 (constant ω rates across all sites) versus M3 (allows ω to vary across sites within *n* discrete categories, where *n* ≥ 3); M1a (a model of neutral evolution) where all sites are assumed to be either under negative (ω <1) or neutral selection (ω = 1) versus M2a (a model of positive selection) which in addition to the site classes mentioned for M1a, assumes a third category of sites; sites with ω >1 (positive selection) and M7 (β) versus M8 (β and ω), and models that mirror the evolutionary constraints of M1 and M2 but assume that ω values are drawn from a β distribution [85]. Only if the alternative models (M3, M2a and M8: allow sites with ω >1) show a better fit in a Likelihood Ratio Test (LRT) relative to their null models (M0, M1a and M7: do not allow sites ω >1), are their results considered significant. LRT is estimated as twice the difference in maximum likelihood values between nested models and compared with the χ^2^ distribution with the appropriate degree of freedom—the difference in the number of parameters between the two models. The Bayes empirical Bayes (BEB) approach [86] was used to identify amino acids under positive selection by calculating the posterior probabilities that a particular amino acid belongs to a given selection class (neutral, conserved, or highly variable). Sites with greater posterior probability (PP ≥ 95%) of belonging to the 'ω > 1 class’ were inferred to be positively selected.

FUBAR [55-57] implemented in HyPhy [78,79] was employed to detect sites evolving under positive and negative selection. MEME [57], which is designed to overcome the drawbacks of site-specific assessments, was used to detect episodic diversifying selection. Mutations were also assessed via a complementary protein-level approach implemented in TreeSAAP [87]. An evolutionary fingerprint analysis was carried out using the ESD algorithm implemented in datamonkey [78,79,88] in order to clearly depict the proportion of sites under different regimes of selection.

Logo plots [54] showing cysteine codon bias were constructed using Geneious software, version 5.4.

### Shiva superfamily phylogenetic tree

The molecular evolutionary history of the Shiva superfamily toxins was reconstructed using Bayesian inference as implemented in MrBayes version 3.2.1 [89], using lset rates = invgamma with the prset aamodelpr = mixed command, which enables the program to optimize between the nine different amino acid substitution matrices implemented in MrBayes. WAG [90] was chosen as the best substitution matrix by the program. Tree searches were run using four Markov chains for a minimum of 10 million generations, sampling every 100th tree. The log likelihood score of each saved tree was plotted against the number of generations to establish the point at which the log-likelihood scores of the analyses reached their asymptote. 25% of the total trees sampled were discarded as burnin. The posterior probabilities for clades were established by constructing a majority rule consensus tree for all trees generated after completion of the burnin. The tree was rooted using the sequence of ω-actinopoditoxin-Mb1a (also known as ω-missulenatoxin-Mb1a) from the Eastern mouse spider *Missulena bradleyi* as an outgroup; this toxin also blocks Ca_V_ channels and it has sequence homology to the ω-HXTXs, including conservation of the ω-HXTX-Hv1a pharmacophore. However, although *M. bradleyi* and Australian funnel-web spiders (family Hexathelidae) both belong to the infraorder Mygalomorphae, *M. bradleyi* is a member of the Actinopodidae family.

### Structural alignment of omega and kappa hexatoxins

Atomic coordinates for ω-HXTX-Hv1a [18] and κ-HXTX-Hv1c [42] were downloaded from the Protein DataBank (PDB accession codes 1AXH and 1DL0, respectively). A structural alignment of the toxins was automatically generated using DaliLite [70]. All structure figures were generated using MacPyMOL [91]. The Consurf webserver [92] was used for mapping evolutionary selection pressures on 3D homology models.

### Availability of supporting data

Nucleic acid and protein sequence alignments and their respective accession numbers can be accessed from the supplementary material along with tables relevant to the molecular evolution analyses.

## Abbreviations

cDNA: Complementary DNA; PolyA+: Polyadenylated RNA; mRNA: Messenger RNA; RACE: Rapid amplification of cDNA ends; PTP: Pico titre plate; AGRF: Australian genome research facility; HXTX: Omega hexatoxins; κ-HXTX: Kappa hexatoxins; H. infensa: *Hadronyche infensa*; H. modesta: *Hadronyche modesta*; H. versuta: *Hadronyche versuta*; H. venenata: *Hadronyche venenata*; A. robustus: *Atrax robustus*; MEME: Mixed effects model evolution; SLAC: Single likelihood ancestor counting; REL: Random effects likelihood; FEL: Fixed-effects likelihood; FUBAR: Fast unbiased approximate bayesian; PAML: Phylogenetic analysis by maximum likelihood; BEB: Bayes empirical Bayes; ASA: Accessible surface area; RAVER: Rapid Accumulation of Variations in Exposed Residues.

## Competing interests

None of the authors have competing interests.

## Authors’ contributions

GFK and BGF proposed the research plan. DW collected spiders. DW and BLS performed venom-gland isolation, cDNA library construction, and RACE analyses. SSP analysed toxin sequences, performed nucleotide and peptide alignments, and submitted sequences to EMBL. BGF, KS, LK and AA performed the molecular evolution analyses. EABU prepared the venom-gland cDNA library from *H. modesta*. All authors were involved in data analysis. GFK, BGF, SSP, and KS wrote the manuscript. All authors read and approved the final manuscript.

## Supplementary Material

Additional file 1 Diversification of a single ancestral gene into a successful toxin superfamily in highly venomous Australian funnel-web spiders. Click here for file
