# Supplementary material for: Diversification of a single ancestral gene into a successful toxin superfamily in highly venomous Australian funnel-web spiders
Source: BMC Genomics. 2014 Mar 5;15:177. doi: 10.1186/1471-2164-15-177 (PMC4029134; doi:10.1186/1471-2164-15-177)
Supplement: Additional file 1 — Diversification of a single ancestral gene into a successful toxin superfamily in highly venomous Australian funnel-web spiders. [file 1471-2164-15-177-S1.PDF]

Supplementary Information

Diversification of a single ancestral gene into a successful toxin superfamily in highly venomous Australian funnel-web spiders

Sandy S. Pineda, Brianna L. Sollod, David Wilson, Aaron Darling, Kartik Sunagar, Eivind A.B. Undheim, Laurence Kely, Agostinho Antunes, Bryan G. Fry, and Glenn F. King

Table 1: Maximum-likelihood parameter estimates for ω-HXTXs

Model	Likelihood (ι)	ω_0[a]	Parameters	Sign.[b]	Sites with ω > 1[c]
					**B.E.B**
**M0 (One ratio)**	-1565.393852	0.64	$= \omega_0$		-
**M1 (Neutral)**	-1548.685108	0.62	P_0: 0.438<br>ω0: 0.14<br>P_1: 0.561<br>ω1: 1.0		-
**M2 (Selection)***	-1547.972110	0.73	P_0: 0.434<br>ω0: 0.16<br>P_1: 0.449<br>ω_1:1.0<br>P_2: 0.115<br>ω_2:1.85	P > 0.05[NS]	0 (PP ≥ 0.99)<br>0 (P ≥ 0.95)
**M3 (Discrete)***	-1546.808533	0.69	P_0: 0.229<br>ω_0: 0.04<br>P_1: 0.536<br>ω1: 0.55<br>P_2: 0.233<br>ω_2: 1.64	P << 0.001	-
**M7 (β)**	-1549.004305	0.58	p: 0.39035<br>q: 0.27423		-
**M8 (β and ω)***	-1547.159647	0.69	p_0: 0.803<br>p: 0.699<br>q: 0.815<br>p_1: 0.196<br>ω: 1.66	P > 0.05[NS]	0 (PP ≥ 0.99)<br>0 (P > 0.95)

Legend

a dn/ds (weighted average)

b Significance of the model in comparison with the null model

c Number of sites with ω > 1 under the Bayes empirical Bayes approach with a posterior probability (PP) more than or equal to 0.99 and 0.95

* Models which allow ω > 1; **P > 0.05[N.S]:** Not significant at 0.05

Table 2: Maximum-likelihood parameter estimates for κ-HXTXs

Model	Likelihood (ι)	ω_0[a]	Parameters	Sign.[b]	Sites with ω > 1[c]
					**B.E.B**
**M0 (One ratio)**	-403.406383	1.06	$= \omega_0$		-
**M1 (Neutral)**	-403.412605	1.0	P_0: 0.00001<br>$\omega0$: 0.0<br>P_1: 0.999<br>$\omega1$:1.0		-
**M2 (Selection)***	-403.406383	1.06	P_0: 0.0<br>ω_0: 1.0<br>P_1: 0.0001<br>ω_1:1.0<br>P_2: 0.999<br>ω_2:1.06	P > 0.05[N.S]	0 (PP ≥ 0.99)<br>0 (P ≥ 0.95)
**M3 (Discrete)***	-403.406383	1.06	P_0: 0.358<br>ω_0: 1.06<br>P_1: 0.352<br>$\omega1$: 1.06<br>P_2: 0.289<br>ω_2: 1.06	P > 0.05[N.S]	-
**M7 (β)**	-403.412606	1.0	p: 0.56027<br>q: 0.00500		-
**M8 (β and ω)***	-403.405829	1.06	p_0: 0.029<br>p: 0.005<br>q: 1.23<br>p1: 0.970<br>ω: 1.09	P > 0.05[N.S]	0 (PP ≥ 0.99)<br>0 (P > 0.95)

Legend

a dn/ds (weighted average)

b Significance of the model in comparison with the null model

c Number of sites with ω > 1 under the Bayes empirical Bayes approach with a posterior probability (PP) more than or equal to 0.99 and 0.95

* Models which allow ω > 1; **P > 0.05[N.S]:** Not significant at 0.05

Table 3: Maximum-likelihood parameter estimates for complete Shiva superfamily (combined)

Model	Likelihood (ι)	ω_0[a]	Parameters	Sign.[b]	Sites with ω > 1[c]
					**B.E.B**
**M0 (One ratio)**	-1863.156921	0.69	$= \omega_0$		-
**M1 (Neutral)**	-1839.163417	0.66	P_0: 0.387<br>ω0: 0.137<br>P_1: 0.612<br>ω1: 1.0		-
**M2 (Selection)***	-1836.952320	0.83	P_0: 0.364<br>ω_0: 0.14<br>P_1: 0.509<br>ω_1: 1.0<br>P_2: 0.125<br>ω_2: 2.15	P > 0.05[NS]	0 (PP ≥ 0.99)<br>0 (P ≥ 0.95)
**M3 (Discrete)***	-1836.534243	0.78	P_0: 0.304<br>ω_0: 0.0<br>P_1: 0.484<br>ω1: 0.75<br>P_2: 0.211<br>ω_2: 1.81	P << 0.001	-
**M7 (β)**	-1840.113352	0.62	p: 0.40011<br>q: 0.24418		-
**M8 (β and ω)***	-1836.795071	0.78	p_0: 0.800<br>p: 0.582<br>q: 0.528<br>p1: 0.199<br>ω: 1.81	P < 0.05	0 (PP ≥ 0.99)<br>1 (P > 0.95)

Legend

a dn/ds (weighted average)

b Significance of the model in comparison with the null model

c Number of sites with ω > 1 under the Bayes empirical Bayes approach with a posterior probability (PP) more than or equal to 0.99 and 0.95

* Models which allow ω > 1; **P > 0.05[N.S]:** Not significant at 0.05

Table 4: **Accession numbers for toxin sequences used in this study**

No.	Toxin Name	Accession number	CODE
1	omega/kappa-hexatoxin-Ar1g_1	HG001293	H
2	omega/kappa-hexatoxinAr1g 2	HG001294	H
3	omega/kappa-hexatoxinAr1g 3	HG001295	H
4	omega/kappa-hexatoxinAr1g 3 2	NA*	H
5	omega/kappa-hexatoxinAr1g_4	HG001296	H
6	omega/kappa-hexatoxinAr1g_5	HG001297	H
7	omega/kappa-hexatoxinHv1g_1	HG001298	H
8	omega/kappa-hexatoxinHv1g 2	HG001299	H
9	omega/kappa-hexatoxinHv1g 3	HG001300	H
10	omega/kappa-hexatoxinHv1g 4	HG001301	H
11	omega/kappa-hexatoxinHv1g_5	HG001302	H
12	omega/kappa-hexatoxinHv1g_6	HG001303	H
13	omega/kappa-hexatoxinHv1g_7	HG001309	H
14	kappa-hexatoxin-Hf1a	P0C2L8	K
15	kappa-hexatoxin-Hv1a	P82227	K
16	kappa-hexatoxin-Hv1b	P82226	K
17	kappa-hexatoxin-Hv1c_1	P82228	K
18	kappa-hexatoxin-Hv1c_2	P82228	K
19	kappa-hexatoxin-Hv1c_3	P82228	K
20	kappa-hexatoxin-Hv1c 4	P82228	K
21	kappa-hexatoxin-Hv1d	HG001311	K
22	kappa-hexatoxin-Hv1e	HG001310	K
23	omega/kappa-hexatoxin-Hv1h	HG001308	H
24	kappa-hexatoxin--Hmo1a	HG001312	K
25	omega-hexatoxin-Ar1a_1	A5AH0, P83580	O
26	omega-hexatoxin-Ar1a 2	A5AH0, P83580	O
27	omega-hexatoxin-Ar1a 3	P83580	O
28	omega-hexatoxin-Ar1b 1	A5A3H1	O
29	omega-hexatoxin-Ar1b_2	A5A3H1	O
30	omega-hexatoxin-Ar1d_1	A5A3H3	O
31	omega-hexatoxin-Ar1d_2	A5A3H3	O
32	omega-hexatoxin-Ar1d 3	A5A3H3	O
33	omega-hexatoxin-Ar1d 4	A5A3H3	O
34	omega-hexatoxin-Ar1e 1	A5A3H4	O
35	omega-hexatoxin-Ar1e 2	A5A3H4	O
36	omega-hexatoxin-Ar1f	A5A3H5	O
37	omega-hexatoxin-Ar1h	NA	O
38	omega-hexatoxin-Hf1a	P0C2L4	O
39	omega-hexatoxin-Hi1a 1	P0C2L5	O
40	omega-hexatoxin-Hi1a 2	P0C2L5	O
41	omega-hexatoxin-Hi1a_2	P0C2L5	O
42	omega-hexatoxin-Hi1a_3	P0C2L5	O
43	omega-hexatoxin-Hi1a_4	P0C2L5	O
44	omega-hexatoxin-Hi1a 5	P0C2L5	O
45	omega-hexatoxin-Hi1b 1	P0C2L6	O
46	omega-hexatoxin-Hi1b 10	P0C2L6	O
47	omega-hexatoxin-Hi1b_2	P0C2L6	O

48	omega-hexatoxin-Hi1b_3	P0C2L6	O
49	omega-hexatoxin-Hi1b_4	P0C2L6	O
50	omega-hexatoxin-Hi1b_5	P0C2L6	O
51	omega-hexatoxin-Hi1b_6	P0C2L6	O
52	omega-hexatoxin-Hi1b_7	P0C2L6	O
53	omega-hexatoxin-Hi1b_8	P0C2L6	O
54	omega-hexatoxin-Hi1b_9	P0C2L6	O
55	omega-hexatoxin-Hi1c_1	P0C2L7	O
56	omega-hexatoxin-Hi1c_2	P0C2L7	O
57	omega-hexatoxin-Hi1c_2_2	P0C2L7	O
58	omega-hexatoxin-Hi1c_3	P0C2L7	O
59	omega-hexatoxin-Hi1d	HG001286	O
60	omega-hexatoxin-Hi1d_2	NA	O
61	omega-hexatoxin-Hi1e	HG001284	O
62	omega-hexatoxin-Hi1f	HG001283	O
63	omega-hexatoxin-Hi1g_1	HG001304	O
64	omega-hexatoxin-Hi1g_2	HG001305	O
65	omega-hexatoxin-Hi1g_3	HG001306	O
66	omega-hexatoxin-Hi1g_4	HG001307	O
67	omega-hexatoxin-Hmo1a_1	HG001313	O
68	omega-hexatoxin-Hmo1a_2	HG001314	O
69	omega-hexatoxin-Hmo1b	HG001315	O
70	omega-hexatoxin-Hmo1c	HG001316	O
71	omega-hexatoxin-Hmo1d_1	HG001317	O
72	omega-hexatoxin-Hmo1d_2	HG001318	O
73	omega-hexatoxin-Hmo1d_3	HG001319	O
74	omega-hexatoxin-Hmo1e	NA	O
75	omega-hexatoxin-Hv1a	P56207	
76	omega-hexatoxin-Hv1b	P81595	O
77	omega-hexatoxin-Hv1c	P81596	O
78	omega-hexatoxin-Hv1d	P81597	O
79	omega-hexatoxin-Hv1e	P81598	O
80	omega-hexatoxin-Hv1f	P81599	O
81	omega-hexatoxin-Hvn1a	HG001285	O
82	omega-hexatoxin-Hvn1a_2	NA	O
83	omega-hexatoxin-Hvn1b_1	HG001287	O
84	omega-hexatoxin-Hvn1b_2	HG001288	O
85	omega-hexatoxin-Hvn1b_3	HG001289	O
86	omega-hexatoxin-Hvn1b_4	HG001290	O
87	omega-hexatoxin-Hvn1b_5	HG001291	O
88	omega-hexatoxin-Hvn1b_6	HG001292	O
89	omega-hexatoxin-Hvn1b_6_2	NA	O
90	omega-actinopodiotoxin	P83588	O

*NA indicates sequences without a corresponding accession number either because they were duplicates (e.g., omega-hexatoxin-Hvn1b_6 and omega-hexatoxin-Hvn1b_6_2) or have an incomplete nucleotide sequence (e.g., omega-hexatoxin-Hmo1e). Toxin sequences can be also be downloaded from the ArachnoServer Spider Toxin database (www.arachnoserver.org).

Figure 1: Alignment of venom-peptide sequences used in this study

```
>omega-hexatoxin-Ar1c          MNTATGVIALLVLATVIGCIEAEDTRADLQG------GEAAEK-VFRRSPTCIPSGQPCPY-NENYCSQ-SCTFKENENANTVKRCD----
>omega-hexatoxin-Ar1h          MNTATGVIALLVLATVIGCIEAEDTRADLQG------GEAAEK-VFRRSPTCIPSGQPCPY-NENCCSQ-SCTFKENENANTVKRCD----
>omega-hexatoxin-Ar1f          MNTATGVIALLVLATVIGCIEAEDTRADLQG------GEAAEK-VFRRSPTCIPSGQPCPY-NENCCSQ-SCTFKENETGNTVKRCD----
>omega-hexatoxin-Ar1d_3        MNTATGVIALLVLATVIGCIEAEDTRADLQG------GEAAEK-VFRRSPTCIPSGQPCPY-NENCCSQ-SCTFKENENGNTVKRCD----
>omega-hexatoxin-Ar1d_2        MNTATGVIALLVLATVIGCIEAEDTRADLQG------GEAAEK-VFRRSPTCIPSGQPCPY-NENCCSQ-SCTFKENENGNTVKRCD----
>omega-hexatoxin-Ar1d_1        MNTATGVIALLVLATVIGCIEAEDTRADLQG------GEAAEK-VFRRSPTCIPSGQPCPY-NENCCSQ-SCTFKENENGNTVKRCD----
>omega-hexatoxin-Ar1d_4        MNTATGVIALLVLVTVIGCIEAEDTRADLQG------GEAAEK-VFRRSPTCIPSGQPCPY-NENCCSQ-SCTFKENENGNTVKRCD----
>omega-hexatoxin-Hvn1a         MNTATGVIALLVLATVIGCIEA-ETRADLQGAFESYEGEAAEK-IFRRSPTCIPSGQPCPY-NENCCSK-SCTYKEMKTATPVQRCD----
>omega-hexatoxin-Hvn1a_2       MNTATGVIALLVLATVIGCIEA-ETRADLQGAFESYEGEAAEK-IFRRSPTCIPSGQPCPY-NENCCSK-SCTYKEMKTATPVQRCD----
>omega-hexatoxin-Hvn1b_3       MNTATGVIALLVLATVIGCIEVEETRADLQGAFESYEGEAAEK-IFRRSPTCIPSGQPCPY-NENCCSK-SCTYKENENGNTVQRCD----
>omega-hexatoxin-Hvn1b_5       MNTATGVIALLVLATVIGCIEAEETRADLQGAFESYEGEAADK-IFRRSPTCIPSGQPCPY-NENCCSK-SCTYKENENGNTVQRCD----
>omega-hexatoxin-Hvn1b_4       MNTATGVIALLVLATVIGCIEAEETRADLQGAFESYEGEAADK-IFRRSPTCIPSGQPCPY-NENCCSK-SCTYKENENGNTVQRCD----
>omega-hexatoxin-Hvn1b_1       MNTATGVIALVVLATVIGCIEAEETRADLQGAFESYEGEAADK-IFRRSPTCIPSGQPCPY-NENCCSK-SCTYKENENGNTVQRCD----
>omega-hexatoxin-Hvn1b_2       MNTATGVIALLVLATVIGCIEAEETRADLQGAFESYEGEGADK-IFRRSPTCIPSGQPCPY-NENCCSK-SCTYKENENGNTVQRCD----
>omega-hexatoxin-Hvn1b_6       MNTATGVIALLVLATVIGCIEAEDTRADLQGGLESYEGEAADK-IFRRSPTCIPSGQPCPY-NENCCSK-SCTYKENENGNTVQRCD----
>omega-hexatoxin-Hvn1b_6_2     MNTATGVIALLVLATVIGCIEAEDTRADLQGGLESYEGEAADK-IFRRSPTCIPSGQPCPY-NENCCSK-SCTYKENENGNTVQRCD----
>omega-hexatoxin-Ar1e_1        MNTATGVIALLVLATVIGCIEAEDTRADLQG------GEAAEK-VFRRSPTCIPSGQPCPY-NENCCSK-SCTYKENENGNTVQRCD----
>omega-hexatoxin-Ar1e_2        MNTATGVIALLVLATVIGCIEAEDTRADLQG------GEAAEK-VFRRSPTCIPSGQPCPY-NENCCSK-SCTYKENENGNTVQRCD----
>omega-hexatoxin-Ar1a_1        MNTATGFIVLLVLATVLGAIEAEDAVPDFEGGFASHAREDTVGGKIRRSSVCIPSGQPCPY-NEHCCSG-SCTYKENENGNTVQRCD----
>omega-hexatoxin-Ar1a_2        MNTATGFIVLLVLATVLGAIEAEDAVPDFEGGFASHAREDTVGGKIRRSSVCIPSGQPCPY-NEHCCSG-SCTYKENENGNTVQRCD----
>omega-hexatoxin-Hmo1d_1       MNTATGVIALLVLATVIGCIQAEDTMADLQGGFESYDGEAAKR-IFRRSPVCIPSGQPCPY-NEHCCSG-SCTYKENENGNTVQRCD----
>omega-hexatoxin-Hmo1d_2       MNTATGVIALLVLATVIGCIQAEDTMADLQGGFESYDGEAAKR-IFRRSPVCIPSGQPCPY-NEHCCSG-SCTYKENENGNTVQRCD----
>omega-hexatoxin-Hmo1d_3       MNTATGVIALLVLATVIGFIEAENTRADLQGGFESYEGEAAEK-IFRRSPVCIPSGQPCPY-NEHCCSG-SCTYKENENGNTVQRCD----
>omega-hexatoxin-Hi1d          MNTATGFIVLLVLATVIGCISA-----DFQGGFEPYEEEDAER-IFRRSPTCIPTGQPCPY-NENCCNQ-SCTYKANENGNQVKRCD----
>omega-hexatoxin-Hi1d_2        MNTATGFIVLLVLATVIGCISA-----DFQGGFEPYEEEDAER-IFRRSPTCIPTGQPCPY-NENCCNQ-SCTYKANENGNQVKRCD----
>omega-hexatoxin-Hi1b_9        MNTATGFIVLLVLATVIGCISA-----DFQGGFEPYEEEDAER-IFRRSPTCIPTGQPCPY-NENCCSQ-SCTYKANENGNQVKRCD----
>omega-hexatoxin-Hi1b_7        MNTATGFIVLLVLATVIGCISA-----DFQGGFEPYEEEDAER-IFRRSPTCIPTGQPCPY-NENCCSQ-SCTYKANENGNQVKRCD----
>omega-hexatoxin-Hi1b_6        MNTATGFIVLLVLATVIGCISA-----DFQGGFEPYEEEDAER-IFRRSPTCIPTGQPCPY-NENCCSQ-SCTYKANENGNQVKRCD----
>omega-hexatoxin-Hi1b_4        MNTATGFIVLLVLATVIGCISA-----DFQGGFEPYEEEDAER-IFRRSPTCIPTGQPCPY-NENCCSQ-SCTYKANENGNQVKRCD----
>omega-hexatoxin-Hi1b_3        MNTATGFIVLLVLATVIGCISA-----DFQGGFEPYEEEDAER-IFRRSPTCIPTGQPCPY-NENCCSQ-SCTYKANENGNQVKRCD----
>omega-hexatoxin-Hi1b_2        MNTATGFIVLLVLATVIGCISA-----DFQGGFEPYEEEDAER-IFRRSPTCIPTGQPCPY-NENCCSQ-SCTYKANENGNQVKRCD----
>omega-hexatoxin-Hi1b_8        MNTATGFIVLLVLATVIGCIST-----DFQGGFEPYEEEDAER-IFRRSPTCIPTGQPCPY-NENCCSQ-SCTYKANENGNQVKRCD----
>omega-hexatoxin-Hi1a_2        MNTATGFIVLLVLATVIGCISA-----DFQGSFEPYEEEDAER-IFRRS-TCTPTDQPCPY-HESCCSG-SCTYKANENGNQVKRCD----
>omega-hexatoxin-Hi1a_4        MNTATGFIVLLVLATVIGCISA-----DFQGSFEPYEEEDAER-IFRRS-TCTPTDQPCPY-HESCCSG-SCTYKANENGNQVKRCD----
>omega-hexatoxin-Hi1a_5        MNTATGFIVLLVLATVIGCISA-----DFQGSFEPYEEEDAER-IFRRS-TCTPTDQPCPY-HESCCSG-SCTYKANENGNQVKRCD----
>omega-hexatoxin-Hi1g_3        MNTATGFIVLLVLATVIGCISA-----DFQGSFEPYEEEDAER-IFRRS-TCTPTDQPCPY-DESCCSG-SCTYKANENGNQVKRCD----
>omega-hexatoxin-Hi1g_4        MNTATGFIVLLVLATVIGCISA-----DFQGSFEPYEEEDAER-IFRRS-TCTPTDQPCPY-DESCCSG-SCTYKANENGNQVKRCD----
>omega-hexatoxin-Hi1g_1        MNTATGFIVLLVLATVIGCISA-----DFEGSFEPYEEEDAER-IFRRS-TCTPTDQPCPY-DESCCSG-SCTYKANENGNQVKRCD----
>omega-hexatoxin-Hi1a_3        MNTATGFIVLLVLATVIGCISA-----DFQGGFEPYEEEDAER-IFRRS-TCTPTDQPCPY-HESCCSG-SCTYKANENGNQVKRCD----
>omega-hexatoxin-Hi1g_2        MNTATGFIVLLVLATVIGCISA-----DFQGGFEPYEEEDAER-IFRRS-TCTPTDQPCPY-DESCCSG-SCTYKANENGNQVKRCD----
>omega-hexatoxin-Hi1c_2        MNTATGFIVLLVLATVIGCISA-----DFQGGFES-SVEDAER-LFRRSSTCIRTDQPCPY-NESCCSG-SCTYKANENGNQVKRCD----
>omega-hexatoxin-Hi1c_2_2      MNTATGFIVLLVLATVIGCISA-----DFQGGFES-SVEDAER-LFRRSSTCIRTDQPCPY-NESCCSG-SCTYKANENGNQVKRCD----
>omega-hexatoxin-Hi1c_3        MNTATGFIVLLVLATVIGCISA-----DFQGGFES-SVEDAER-LFRRSSTCIRTDQPCPY-NESCCSG-SCTYKANENGNQVKRCD----
>omega-hexatoxin-Hi1b_10       MNTATGFIVLLVLATVIGCISA-----DFQGGFEPYEEEDAQR-IFRRSPTCIPTGQPCPY-NENCCSQ-SCTYKANENGNQVKRCD----
>omega-hexatoxin-Hi1f          MNTATGFIVLLVLATVIGCISA-----DFQGGFEPYEEEDAER-IFRRSPTCIPTGQPCPY-NENCCSQ-SCTYKTNENGNQVKGCD----
>omega-hexatoxin-Hi1e          MNTATGFIVLLVLATVIGCISA-----DFQGGFEPYEGEDAER-IFRRSPTCIPTGQPCPY-NENCCSQ-SCTYKANENGNQVKGCD----
>omega-hexatoxin-Hi1b_5        MNTATGFIVLLVLATVIGCISV-----DFQGGFESYEEEDAER-IFRRSPTCIPTGQPCPY-NENCCSQ-SCTYKANENGNQVKRCD----
>omega-hexatoxin-Hmo1a_1       MNTATGVIALLVLATVIGCIEAEDTREDFQGGFESYDGEAAEK-IFRRAPVCTRTDQPCPY-NEDCCSG-SCTLKKNENGNLVKRCD----
```

```
>omega-hexatoxin-Hmo1b          MNTATGVIALLVLATVIGCIEAEDTREDFQGGFESDDGEAAEK-IFRRAPVCTRTDQPCPY-DQDCCSG-SCTLKKNENGNLVKRCD----
>omega-hexatoxin-Hmo1a_2        MNTATGVIVLLVLATVIGCIEAEDTREDLQGGFESYDGEAAEK-IFRRAPVCTRTDQPCPY-NEDCCSG-SCTLKKNENGNLVKRCD----
>omega-hexatoxin-Hmo1c          MNTATGVIALLVLATVIGFIEAENTRADLQGGFESYEGEAAEK-IFRRSPVCTRTDQPCPY-DQDCCSG-SCTLKKNENGNLVKRCD----
>omega-hexatoxin-Ar1b_1         MNTATGFIVLLVLATVLGCIEA--------G--ESHVREDAMG-RARR-GACTPTGQPCPY-NESCCSG-SCQEQLNENGHTVKRCV----
>omega-hexatoxin-Ar1b_2         MNTATGFIVLLVLATVLGCIEA--------G--ESHVREDAMG-RARR-GACTPTGQPCPY-NESCCSG-SCQEQLNENGHTVKRCV----
>omega-hexatoxin-Hv1a           ------------------------------------------------SPTCIPSGQPCPY-NENCCSQ-SCTFKENENGNTVKRCD----
>omega-hexatoxin-Hi1b_1         ------------------------------------------------SPTCIPTGQPCPY-NENCCSQ-SCTYKANENGNQVKRCD----
>omega-hexatoxin-Hf1a           ------------------------------------------------SPTCIRSGQPCPY-NENCCSQ-SCTFKTNENGNTVKRCD----
>omega-hexatoxin-Hv1c           ------------------------------------------------SSTCIPSGQPCPY-NENCCSQ-SCTFKENENGNTVKRCD----
>omega-hexatoxin-Hv1b           ------------------------------------------------SSTCIPSGQPCPY-NENCCSQ-SCTYKENENGNTVKRCD----
>omega-hexatoxin-Hv1e           ------------------------------------------------SPTCIPSGQPCPY-NENCCSQ-SCTYKENENGNTVKRCD----
>omega-hexatoxin-Hv1d           ------------------------------------------------SPTCIPSGQPCPY-NENCCSK-SCTYKENENGNTVQRCD----
>omega-hexatoxin-Ar1a_3         ------------------------------------------------SSVCIPSGQPCPY-NEHCCSG-SCTYKENENGNTVQRCD----
>omega-hexatoxin-Hv1f           ------------------------------------------------SAVCIPSGQPCPY-SKYCCSG-SCTYKTNENGNSVQRCD----
>omega-hexatoxin-Hmo1e          ------------------------------------------------STVCIPSGQPCPY-SKYCCSG-SCTYKENENGNTVQRCD----
>omega-hexatoxin-Hi1a_1         ------------------------------------------------ST-CTPTDQPCPY-HESCCSG-SCTYKANENGNQVKRCD----
>omega-hexatoxin-Hi1c_1         ------------------------------------------------SSTCIRTDQPCPY-NESCCSG-SCTYKANENGNQVKRCD----
>omega/kappa-hexatoxin-Ar1g_1   MNTATGFIVLLVLATVLGGIEA--------G--ESHMRKDAMG-RVRR-QYCVPVDQPCSLNTQPCCDDATCTQELNENDNTVYYCRA---
>omega/kappa-hexatoxin-Ar1g_2   MNTATGFIVLLVLATVLGGIEA--------R--ESHMRKDAMG-RVRR-QYCVPVDQPCSLNTQPCCDDATCTQELNENDNTVYYCRA---
>omega/kappa-hexatoxin-Ar1g_3   MNTATGFIVLLVLATVLGGIEA--------G--ESHMRKDAMG-RVRR-QYCVPVDQPCSLNTQPCCDDATCTQELNENDNTVYYCRA---
>omega/kappa-hexatoxin-Ar1g_3_2 MNTATGFIVLLVLATVLGGIEA--------G--ESHMRKDAMG-RVRR-QYCVPVDQPCSLNTQPCCDDATCTQELNENDNTVYYCRA---
>omega/kappa-hexatoxin-Ar1g_4   MNTATGFIVLLVLATVLGGIEA--------G--ESHMRKDAMG-RVRR-QYCVPVDQPCSLNTQPCCDDATCTQELNENDNTVYYCRA---
>omega/kappa-hexatoxin-Ar1g_5   MNTATGFIVLLVLATVLGGIEA--------G--ESHMRKDAMG-RVRR-QYCVPVDQPCSLNTQPCCDDATCTQELNENDNTVYYCRA---
>omega/kappa-hexatoxin-Hv1g_1   MNTATGFIVLLVLATVLGGIEA--------G--ESHMRKDAMG-RVRR-QYCVPVDQPCSLNTQPCCDDATCTQELNENDNTVYYCRA---
>omega/kappa-hexatoxin-Hv1g_2   MNTATGFIVLLVLATVLGGIEA--------G--ESHMRKDAMG-RVRR-QYCVPVDQPCSLNTQPCCDDATCTQELNENDNTVYYCRA---
>omega/kappa-hexatoxin-Hv1g_3   MNTATGFIVLLVLATVLGGIEAG----------ESHMRKDA-MGRVRR-QYCVPVDQPCSLNTQPCCDDATCTQELNENDNTVYYCRA---
>omega/kappa-hexatoxin-Hv1g_4   MNTATGFIVLLVLATVLGGIEA--------G--ESHMRKDAMG-RVRR-QYCVPVDQPCSLNTQPCCDDATCTQELNENDNTVYYCRA---
>omega/kappa-hexatoxin-Hv1g_5   MNTTTGFIVLLVLATILGGIEA--------G--ESHMRKDAMG-RVRR-QYCVPVDQPCSLNTQPCCDDATCTQELNENDNTVYYCRA---
>omega/kappa-hexatoxin-Hv1g_6   MNTATGFIVFLVLATVLGGIEA--------G--ESHMRKDAMG-RVRR-QYCVPVDQPCSLNTQPCCDDATCTQELNENDNTVYYCRA---
>omega/kappa-hexatoxin-Hv1g_7   -NTATGFIVLLVLATVLGGIEA--------G--ESHMRKDAMG-RVRR-QYCVPVDQPCSLNTQPCCDDATCTQELNENDNTVY-------
>omega/kappa-hexatoxin-Hv1h     MNTATGFIVLLVLATILGGIEA--------G--ESHMRKDAMG-RVRR-QYCVPVDQPCSLNTQPCCDDATCTQERNENGHTVYYCRA---
>Kappa-hexatoxin-Hv1a           -------------------------------------------------TICTGADRPCAA-CCPCCPGTSCQG---PESNGVVYCRNF--
>Kappa-hexatoxin-Hv1b           -------------------------------------------------TICTGADRPCAA-CCPCCPGTSCQG---PEPNGVSYCRND--
>Kappa-hexatoxin-Hf1a           ------------------------------------------------SPTCTGADRPCAA-CCPCCPGTSCKG---PEPNGVSYCRND--
>Kappa-hexatoxin-Hv1c_1         -------------------------------------------------AICTGADRPCAA-CCPCCPGTSCK----AESNGVSYCRKDEP
>Kappa-hexatoxin-Hv1c_3         MNTATCFIVLLVVATVIGGIEA--------G--EFDMRKDVMG-LFRR-AICTGADRPCAA-CCPCCPGTSCK----AESNGVSYCRKDEP
>Kappa-hexatoxin-Hv1c_2         MNTATCFIVLLDVATVIGGIEA--------G--ESDMRKDVMG-LFRR-AICTGADRPCAA-CCPCCPGTSCK----AESNGVSYCRKDEP
>Kappa-hexatoxin-Hv1c_4         MNTATCFIVFLVVATVIGGIEA--------G--ESDMRKDVMG-LFRR-AICTGADRPCAA-CCPCCPGTSCK----AESNGVSYCRKDEP
>Kappa-hexatoxin-Hv1e           MNTATCFIVLLVVATVIGGIEA--------G--EFDMRKDVMG-LFRR-AICPGADRPCAA-CCPCCPGTSCK----AESNGVFYCRKDEP
>Kappa-hexatoxin-Hv1d           MNTATCFIVLLVVATVIGGIEA--------G--ESDMRKDVMG-LFRR-VICTGADSPCAA-CCPCCPGTSCK----AESNGVSYCRKDEP
>kappa-hexatoxin-Hmo1a          MNTATGFIVLLVXATVIGGIEA--------G--ESDMRKDV-MGLFRR-TICTGADRPCAA-CCPCCPGTSC-QGP--ESNXISYCRND--
>omega-actinopoditoxin-Mb1a     ------------------------------------------------SPVCTPSGQPCQPNTQPCCNNAEEEQTINCNGNTVYRCA----
```

Figure 2: Alignment of nucleotide sequences used in this study

```
>omega-hexatoxin-Hmo1a_2
ATGAATACTGCTACAGGTGTCATCGTTCTTTTGGTGTTGGCGACAGTCATCGGATGCATTGAAGCAGAAGATACCAGGGAAGATCTTCAAGGAGGTTTCGAATCTTATGACGGAGAAGCCGCCGAGAAAATA---
TTTCGCCGGGCCCCGGTTTGCACTCGAACTGATCAACCGTGCCCC---TACAACGAAGATTGCTGCAGCGGTTCC---TGTACACTTAAGAAAAATGAAAACGGCAACTTAGTTAAAAGATGC------GAC------
>omega-heatoxin-Hmo1d_1
ATGAATACCGCTACAGGTGTCATCGCTCTTTTGGTTCTGGCGACAGTCATCGGATGCATTCAAGCAGAAGATACCATGGCAGATCTTCAAGGAGGTTTCGAATCTTATGACGGAGAAGCCGCCAAGAGAATA---
TTTCGCCGCTCCCCGGTTTGCATTCCATCTGGTCAACCATGCCCC---TACAACGAACATTGCTGCAGCGGTTCC---TGTACATATAAGGAAAATGAAAACGGCAACACTGTTCAAAGATGC------GAC------
>omega-hexatoxin-Hmo1b
ATGAATACCGCTACAGGTGTCATCGCTCTTTTGGTTCTGGCGACAGTCATCGGATGCATTGAAGCAGAAGATACCAGGGAAGATTTTCAAGGAGGTTTCGAATCTGATGACGGAGAAGCCGCCGAGAAAATA---
TTTCGCCGGGCCCCGGTTTGCACTCGAACTGATCAACCGTGCCCC---TACGACCAAGATTGTTGCAGCGGTTCC---TGTACACTTAAGAAAAATGAAAACGGCAACTTAGTTAAAAGATGC------GAC------
>omega-hexatoxin-Hmo1d_2
ATGAATACCGCTACAGGTGTCATCGCTCTTTTGGTTCTGGCGACAGTCATCGGATGCATTCAAGCAGAAGATACCATGGCAGATCTTCAAGGAGGTTTCGAATCTTATGACGGAGAAGCCGCCAAGAGAATA---
TTTCGCCGCTCCCCGGTTTGCATTCCATCTGGTCAACCATGCCCC---TACAACGAACATTGCTGCAGCGGTTCC---TGTACATATAAGGAAAATGAAAACGGCAACACTGTTCAAAGATGC------GAC------
>omega-hexatoxin-Hmo1d_3
ATGAATACCGCTACAGGTGTCATCGCTCTTTTGGTTCTGGCGACAGTCATCGGATTCATTGAAGCAGAAAATACCAGGGCAGATCTACAAGGAGGTTTCGAATCTTATGAAGGAGAAGCCGCCGAGAAAATA---
TTTCGCCGCTCCCCGGTTTGCATTCCATCTGGTCAACCATGCCCC---TACAACGAACATTGCTGCAGCGGTTCC---TGTACATATAAGGAAAATGAAAACGGCAACACTGTTCAAAGATGC------GAC------
>omega-hexatoxin-Hmo1a_1
ATGAATACCGCTACAGGTGTCATCGCTCTTTTGGTTCTGGCGACAGTCATCGGATGCATTGAAGCAGAAGATACCAGGGAAGATTTTCAAGGAGGTTTCGAATCTTATGACGGAGAAGCCGCCGAGAAAATA---
TTTCGCCGGGCCCCGGTTTGCACTCGAACTGATCAACCCTGCCCC---TACAACGAAGATTGCTGCAGCGGTTCC---TGTACACTTAAGAAAAATGAAAACGGCAACTTAGTTAAAAGATGC------GAC------
>omega-hexatoxin-Hmo1c
ATGAATACCGCTACAGGTGTCATCGCTCTTTTGGTTCTGGCGACAGTCATCGGATTCATTGAAGCAGAAAATACCAGGGCAGATCTACAAGGAGGTTTCGAATCTTATGAAGGAGAAGCCGCCGAGAAAATA---
TTTCGCCGCTCCCCGGTTTGCACTCGAACTGATCAACCGTGCCCC---TACGACCAAGATTGTTGCAGCGGTTCC---TGTACACTTAAGAAAAATGAAAACGGCAACTTAGTTAAAAGATGC------GAC------
>omega-hexatoxin-Hmo1e
---------------------------------------------------------------------------------------------------------------------------------------------
TCCACGGTTTGCATTCCTTCTGGTCAACCATGCCCA---TACAGTAAATATTGCTGCAGCGGTTCC---TGTACATATAAGGAAAATGAAAACGGCAACACTGTTCAAAGATGC------GAC------
>omega-hexatoxin-Hi1a_2_2
ATGAATACCGCTACAGGTTTCATCGTACTTTTGGTTTTGGCGACAGTGATCGGATGCATTTCTGCA---------------GATTTTCAAGGAAGTTTCGAACCC------------------GAAAGAATA---
TTTCGCCGCTCAACT---TGCACTCCAACTGATCAACCGTGCCCC---TACCACGAAAGTTGCTGCAGCGGTTCC---TGTACATATAAGGCAAATGAAAACGGCAACCAAGTTAAAAGATGC------GAC------
>omega-hexatoxin-Hi1d_2
ATGAATACCGCTACAGGTTTCATCGTACTTTTGGTTTTGGCGACAGTGATCGGATGCATTTCTGCA---------------GATTTTCAAGGAGGTTTCGAACCTTATGAAGAAGAAGACGCCGAAAGAATA---
TTTCGCCGCTCCCCAACTTGCATTCCAACTGGTCAACCGTGTCCC---TACAACGAAAATTGCTGCAACCAATCC---TGTACATATAAGGCAAATGAAAACGGCAACCAAGTTAAAAGATGC------GAC------
>omega-hexatoxin-Hvn1b_6_2 A
ATGAATACTGCTACAGGTGTCATCGCTCTTTTGGTTCTGGCGACAGTCATCGGATGCATTGAAGCAGAAGATACCAGAGCAGATCTTCAAGGAGGTTTGGAATCTTATGAAGGAGAAGCCGCCGATAAAATA---
TTCCGCCGCTCCCCGACTTGCATTCCATCTGGTCAACCATGCCCC---TACAACGAAAATTGCTGCAGCAAATCG---TGTACATATAAGGAAAATGAAAACGGCAACACTGTTCAAAGATGC------GAC------
>omega-hexatoxin-Ar1a_1
ATGAATACCGCAACAGGTTTCATCGTCCTTTTGGTTTTGGCGACAGTTCTCGGAGCCATTGAAGCAGAAGATGCCGTGCCAGATTTTGAAGGAGGTTTCGCATCTCATGCAAGAGAAGACACCGTAGGAGGAAAAATTCGCCGCTCC
TCGGTTTGCATTCCATCTGGTCAGCCGTGCCCG---TACAACGAACATTGCTGCAGTGGTTCA---TGTACATACAAGGAAAATGAAAACGGCAACACTGTTCAAAGATGC------GAC------
>omega-hexatoxin-Ar1a_2
ATGAATACCGCAACAGGTTTCATCGTCCTTTTGGTTTTGGCGACAGTTCTCGGAGCCATTGAAGCAGAAGATGCCGTGCCAGATTTTGAAGGAGGTTTCGCATCTCATGCAAGAGAAGACACCGTAGGAGGAAAAATTCGCCGCTCC
TCGGTTTGCATTCCATCTGGTCAGCCGTGCCCG---TACAACGAACATTGCTGCAGTGGTTCA---TGTACATACAAGGAAAATGAAAACGGCAACACTGTTCAAAGATGC------GAC------
>omega-hexatoxin-Ar1b_1
ATGAATACCGCAACAGGTTTCATTGTCCTTTTGGTTTTGGCGACAGTTCTTGGATGCATTGAAGCAGGAGAATCTCATGTGAGAGAAGACGCC-----------------------------------
ATGGGAAGAGCTCGCCGGGGGGCT---TGCACTCCAACTGGTCAACCGTGCCCG---TATAACGAAAGTTGTTGCAGCGGTTCC---TGCCAAGAACAGCTAAATGAAAACGGACACACTGTTAAAAGATGCGTT------------
>omega-hexatoxin-Ar1b_2
ATGAATACCGCAACAGGTTTCATCGTCCTTTTGGTTTTGGCGACAGTTCTTGGATGCATTGAAGCAGGAGAATCTCATGTGAGAGAAGACGCC-----------------------------------
ATGGGAAGAGCTCGCCGGGGGGCT---TGCACTCCAACTGGTCAACCGTGCCCG---TATAACGAAAGTTGTTGCAGCGGTTCC---TGCCAAGAACAGCTAAATGAAAACGGCCACACCGTTAAAAGATGCGTT------------
>omega-hexatoxin-Ar1c
ATGAATACCGCTACAGGTGTCATCGCTCTTTTGGTTCTGGCGACAGTCATCGGATGCATTGAAGCAGAAGATACCAGAGCAGATCTTCAAGGAGGAGAAGCC------GCC---GAG---AAAGTA---------
TTTCGCCGCTCCCCGACTTGCATTCCATCTGGTCAACCATGTCCC---TACAACGAAAATTACTGCAGCCAATCG---TGTACATTTAAGGAAAATGAAAACGCCAACACTGTTAAAAGATGC------GAC------
```

>omega-hexatoxin-Ar1d_1
ATGAATACCGCTACAGGTGTCATCGCTCTTTTGGTTCTGGCGACAGTCATCGGATGCATTGAAGCAGAAGATACCAGAGCAGATCTTCAAGGAGGAGAAGCC------GCC---GAG---AAAGTA---------
TTTCGCCGCTCCCCGACTTGCATTCCATCTGGTCAACCATGTCCC---TACAACGAAAATTGCTGCAGCCAATCG---TGTACATTTAAGGAAAATGAAAACGGCAACACTGTTAAAAGATGC------GAC------
>omega-hexatoxin-Ar1d_2
ATGAATACCGCTACAGGTGTCATCGCTCTTTTGGTTCTGGCGACAGTCATCGGATGCATTGAAGCAGAAGATACCAGAGCAGATCTTCAAGGAGGAGAAGCC------GCC---GAG---AAAGTA---------
TTTCGCCGCTCCCCGACTTGCATTCCATCTGGTCAACCATGTCCC---TACAACGAAAATTGCTGCAGCCAATCG---TGTACATTTAAGGAAAATGAAAACGGCAACACTGTTAAAAGATGC------GAC------
>omega-hexatoxin-Ar1d_3
ATGAATACCGCTACAGGTGTCATCGCTCTTTTGGTTCTGGCGACAGTCATCGGATGCATTGAAGCAGAAGATACCAGAGCAGATCTTCAAGGAGGAGAAGCC------GCC---GAG---AAAGTA---------
TTTCGCCGCTCCCCGACTTGCATTCCATCTGGTCAACCATGTCCC---TACAACGAAAATTGCTGCAGCCAATCG---TGTACATTTAAGGAAAATGAAAACGGCAACACTGTTAAAAGATGC------GAC------
>omega-hexatoxin-Ar1d_4
ATGAATACCGCTACAGGTGTCATCGCTCTTTTGGTTCTGGTGACAGTCATCGGATGCATTGAAGCAGAAGATACCAGAGCAGATCTTCAAGGAGGAGAAGCC------GCC---GAG---AAAGTA---------
TTTCGCCGCTCCCCGACTTGCATTCCATCTGGTCAACCATGTCCC---TACAACGAAAATTGCTGCAGCCAATCG---TGTACATTTAAGGAAAATGAAAACGGCAACACTGTTAAAAGATGC------GAC------
>omega-hexatoxin-Ar1e_1
ATGAATACCGCTACAGGTGTCATCGCTCTTTTGGTTCTGGCGACAGTCATCGGATGCATTGAAGCAGAAGATACCAGAGCAGATCTTCAAGGAGGAGAAGCC------GCC---GAG---AAAGTA---------
TTTCGCCGCTCCCCGACTTGCATTCCATCTGGTCAACCATGTCCC---TACAACGAAAATTGCTGCAGCAAATCG---TGTACATATAAGGAAAATGAAAATGGCAACACTGTTCAAAGATGC------GAC------
>omega-hexatoxin-Ar1e_2
ATGAATACCGCTACAGGTGTCATCGCTCTTTTGGTTCTGGCGACAGTCATCGGATGCATTGAAGCAGAAGATACCAGAGCAGATCTTCAAGGAGGAGAAGCC------GCC---GAG---AAAGTA---------
TTTCGCCGCTCCCCGACTTGCATTCCATCTGGTCAACCATGTCCC---TACAACGAAAATTGCTGCAGCAAATCG---TGTACATATAAGGAAAATGAAAACGGCAACACTGTTCAAAGATGC------GAC------
>omega-hexatoxin-Ar1f
ATGAATACCGCTACAGGTGTCATCGCTCTTTTGGTTCTGGCGACAGTCATCGGATGCATTGAAGCAGAAGATACCAGAGCAGATCTTCAAGGAGGAGAAGCC------GCC---GAG---AAAGTA---------
TTTCGCCGCTCCCCGACTTGCATTCCATCTGGTCAACCATGTCCC---TACAACGAAAATTGCTGCAGCCAATCG---TGTACATTTAAGGAAAATGAAACCGGCAACACTGTTAAAAGATGC------GAC------
>omega-hexatoxin-Hi1a_2
ATGAATACCGCTACAGGTTTCATCGTACTTTTGGTTTTGGCGACAGTGATCGGATGCATTTCTGCA---GATTTTCAAGGAAGTTTCGAACCC---GAA---------------AGAATA---------------
TTTCGCCGCTCAACT---TGCACTCCAACTGATCAACCGTGCCCC---TACCACGAAAGTTGCTGCAGCGGTTCC---TGTACATATAAGGCAAATGAAAACGGCAACCAAGTTAAAAGATGC------GAC------
>omega-hexatoxin-Hi1a_3
ATGAATACCGCTACAGGTTTCATCGTACTTTTGGTTTTGGCGACAGTGATCGGATGCATTTCTGCA---GATTTTCAAGGAGGTTTCGAACCTTATGAAGAAGAAGACGCCGAAAGAATA---------------
TTTCGCCGCTCAACT---TGCACTCCAACTGATCAACCGTGCCCC---TACCACGAAAGTTGCTGCAGCGGTTCC---TGTACATATAAGGCAAATGAAAACGGCAACCAAGTTAAAAGATGC------GAC------
>omega-hexatoxin-Hi1a_4
ATGAATACTGCTACAGGTTTCATCGTACTTTTGGTTTTGGCGACAGTGATCGGATGCATTTCTGCA---GATTTTCAAGGAAGTTTCGAACCTTATGAAGAAGAAGACGCCGAAAGAATA---------------
TTTCGCCGCTCAACT---TGCACTCCAACTGATCAACCGTGCCCC---TACCACGAAAGTTGCTGCAGCGGTTCC---TGTACATATAAGGCAAATGAAAACGGCAACCAAGTTAAAAGATGC------GAC------
>omega-hexatoxin-Hi1a_5
ATGAATACTGCTACAGGTTTCATCGTACTTTTGGTTTTGGCGACAGTGATCGGATGCATTTCTGCA---GATTTTCAAGGAAGTTTCGAACCTTATGAAGAAGAAGACGCCGAAAGAATA---------------
TTTCGCCGCTCAACT---TGCACTCCAACTGATCAACCGTGCCCC---TACCACGAAAGTTGCTGCAGCGGTTCC---TGTACATATAAGGCAAATGAAAACGGCAACCAAGTTAAAAGATGC------GAC------
>omega-hexatoxin-Hi1b_2
ATGAATACCGCTACAGGTTTCATCGTACTTTTGGTTTTGGCGACAGTGATCGGATGCATTTCTGCA---GATTTTCAAGGAGGTTTCGAACCTTATGAAGAAGAAGACGCCGAAAGAATA---------------
TTTCGCCGCTCCCCAACTTGCATTCCAACTGGTCAACCGTGTCCC---TACAACGAAAATTGCTGCAGCCAATCC---TGTACATATAAGGCAAATGAAAACGGCAACCAAGTTAAAAGATGC------GAC------
>omega-hexatoxin-Hi1b_3
ATGAATACCGCTACAGGTTTCATCGTACTTTTGGTTTTGGCGACAGTGATCGGATGCATTTCTGCA---GATTTTCAAGGAGGTTTCGAACCTTATGAAGAAGAAGACGCCGAAAGAATA---------------
TTTCGCCGCTCCCCAACTTGCATTCCAACTGGTCAACCGTGTCCC---TACAACGAAAATTGCTGCAGCCAATCC---TGTACATATAAGGCAAATGAAAACGGCAACCAAGTTAAAAGATGC------GAC------
>omega-hexatoxin-Hi1b_4
ATGAATACCGCTACAGGTTTCATCGTACTTTTGGTTTTGGCGACAGTGATCGGATGCATTTCTGCA---GATTTTCAAGGAGGTTTCGAACCTTATGAAGAAGAAGACGCCGAAAGAATA---------------
TTTCGCCGCTCCCCAACTTGCATTCCAACTGGTCAACCGTGTCCC---TACAACGAAAATTGCTGCAGCCAATCC---TGTACATATAAGGCAAATGAAAACGGCAACCAAGTTAAAAGATGC------GAC------
>omega-hexatoxin-Hi1b_5
ATGAATACCGCTACAGGTTTCATCGTACTTTTGGTTTTGGCGACAGTGATCGGATGTATTTCTGTA---GATTTTCAAGGAGGTTTCGAATCTTATGAAGAAGAAGACGCCGAAAGAATA---------------
TTTCGCCGCTCCCCAACTTGCATTCCAACTGGTCAACCGTGTCCC---TACAACGAAAATTGCTGCAGCCAATCC---TGTACATATAAGGCAAATGAAAACGGCAACCAAGTTAAAAGATGC------GAC------
>omega-hexatoxin-Hi1b_6
ATGAATACCGCTACAGGTTTCATCGTTCTTTTGGTTTTGGCGACAGTGATCGGATGCATTTCTGCA---GATTTTCAAGGAGGTTTCGAACCTTATGAAGAAGAAGACGCCGAAAGAATA---------------
TTTCGCCGCTCCCCAACTTGCATTCCAACTGGTCAACCGTGTCCC---TACAACGAAAATTGCTGCAGCCAATCC---TGTACATATAAGGCAAATGAAAACGGCAACCAAGTTAAAAGATGC------GAC------
>omega-hexatoxin-Hi1b_7
ATGAATACTGCTACAGGTTTCATCGTACTTTTGGTTTTGGCGACAGTGATCGGATGTATTTCTGCA---GATTTTCAAGGAGGTTTCGAACCTTATGAAGAAGAAGACGCCGAAAGAATA---------------
TTTCGCCGCTCCCCAACTTGCATTCCAACTGGTCAACCGTGTCCC---TACAACGAAAATTGCTGCAGCCAATCC---TGTACATATAAGGCAAATGAAAACGGCAACCAAGTTAAAAGATGC------GAC------

>omega-hexatoxin-Hi1b_8
ATGAATACTGCTACAGGTTTCATCGTACTTTTGGTTTTGGCGACAGTGATCGGATGCATTTCTACA---GATTTTCAAGGAGGTTTCGAACCTTATGAAGAAGAAGACGCCGAAAGAATA---------------
TTTCGCCGCTCCCCAACTTGCATTCCAACTGGTCAACCGTGTCCC---TACAACGAAAATTGCTGCAGCCAATCC---TGTACATATAAGGCAAATGAAAACGGCAACCAAGTTAAAAGATGC------GAC------
>omega-hexatoxin-Hi1b_9
ATGAATACTGCTACAGGTTTCATCGTACTTTTGGTTTTGGCGACAGTGATCGGATGCATTTCTGCA---GATTTTCAAGGAGGTTTCGAACCTTATGAAGAAGAAGACGCCGAAAGAATA---------------
TTTCGCCGCTCCCCAACTTGCATTCCAACTGGTCAACCGTGTCCC---TACAACGAAAATTGCTGCAGCCAATCC---TGTACATATAAGGCAAATGAAAACGGCAACCAAGTTAAAAGATGC------GAC------
>omega-hexatoxin-Hi1b_10
ATGAATACTGCTACAGGTTTCATCGTACTTTTGGTTTTGGCGACAGTGATCGGATGCATTTCTGCA---GATTTTCAAGGAGGTTTCGAACCTTATGAAGAAGAAGACGCCCAAAGGATA---------------
TTTCGCCGCTCCCCAACTTGCATTCCAACTGGTCAACCGTGTCCC---TACAACGAAAATTGCTGCAGCCAATCC---TGTACATATAAGGCAAATGAAAACGGCAACCAAGTTAAAAGATGC------GAC------
>omega-hexatoxin-Hi1c_2
ATGAATACCGCTACAGGTTTCATCGTTCTTTTGGTTTTGGCGACAGTGATCGGATGCATTTCTGCA---GATTTTCAAGGAGGTTTCGAATCTTCTGTAG---AAGACGCCGAAAGATTA---------------
TTTCGCCGCTCCTCAACTTGCATTCGAACTGATCAACCGTGCCCC---TACAACGAAAGTTGCTGCAGCGGTTCC---TGTACATATAAGGCAAATGAAAACGGAAACCAAGTTAAAAGATGC------GAC------
>omega-hexatoxin-Hi1c_3
ATGAATACCGCTACAGGTTTCATCGTTCTTTTGGTTTTGGCGACAGTGATCGGATGCATTTCTGCA---GATTTTCAAGGAGGTTTCGAATCTTCTGTAG---AAGACGCCGAAAGATTA---------------
TTTCGCCGCTCCTCAACTTGCATTCGAACTGATCAACCGTGCCCC---TACAACGAAAGTTGCTGCAGCGGTTCC---TGTACATATAAGGCAAATGAAAACGGCAACCAAGTTAAAAGATGC------GAC------
>omega-hexatoxin-Hi1f
ATGAATACCGCTACAGGTTTCATCGTACTTTTGGTTTTGGCGACAGTGATCGGATGTATTTCTGCA---GATTTTCAAGGAGGTTTCGAACCTTATGAAGAAGAAGACGCCGAAAGAATA---------------
TTTCGCCGCTCCCCAACTTGCATTCCAACTGGTCAACCGTGTCCC---TACAACGAAAATTGCTGCAGCCAATCC---TGTACATATAAGACAAATGAAAACGGCAACCAAGTTAAAGGATGC------GAC------
>omega-hexatoxin-Hi1e
ATGAATACCGCTACAGGTTTCATCGTACTTTTGGTTTTGGCGACAGTGATCGGATGCATTTCTGCA---GATTTTCAAGGAGGTTTCGAACCTTATGAAGGAGAAGACGCCGAAAGAATA---------------
TTTCGCCGCTCCCCAACTTGCATTCCAACTGGTCAACCGTGTCCC---TACAACGAAAATTGCTGCAGCCAATCC---TGTACATATAAGGCAAATGAAAACGGCAACCAAGTTAAAGGATGC------GAC------
>omega-hexatoxin-Hvn1a
ATGAATACCGCTACAGGTGTCATCGCTCTTTTGGTTCTGGCGACAGTCATCGGATGCATTGAAGCAGAA---ACCAGAGCAGATCTTCAAGGAGCTTTCGAATCTTATGAAGGAGAAGCCGCCGAGAAAATA---
TTTCGCCGCTCCCCGACTTGCATTCCATCTGGTCAACCATGCCCC---TACAACGAAAATTGCTGCAGCAAATCG---TGTACATATAAGGAAATGAAAACGGCAACACCTGTTCAAAGATGC------GAC------
>omega-hexatoxin-Hi1d
ATGAATACCGCTACAGGTTTCATCGTACTTTTGGTTTTGGCGACAGTGATCGGATGCATTTCTGCA---GATTTTCAAGGAGGTTTCGAACCTTATGAAGAAGAAGACGCCGAAAGAATA---------------
TTTCGCCGCTCCCCAACTTGCATTCCAACTGGTCAACCGTGTCCC---TACAACGAAAATTGCTGCAACCAATCC---TGTACATATAAGGCAAATGAAAACGGCAACCAAGTTAAAAGATGC------GAC------
>omega-hexatoxin-Hvn1b_1
ATGAATACCGCTACAGGTGTCATCGCTCTTGTGGTTCTGGCGACAGTCATCGGATGCATTGAAGCAGAAGAAACCAGAGCAGATCTTCAAGGAGCTTTCGAATCTTATGAAGGAGAAGCCGCCGATAAAATA---
TTTCGCCGCTCCCCGACTTGCATTCCATCTGGTCAACCATGCCCC---TACAACGAAAATTGCTGCAGCAAATCG---TGTACATATAAGGAAAATGAAAACGGCAACACTGTTCAAAGATGC------GAC------
>omega-hexatoxin-Hvn1b_2
ATGAATACCGCTACAGGTGTCATCGCTCTTTTGGTTCTGGCGACAGTCATCGGATGCATTGAAGCAGAAGAAACCAGAGCAGATCTTCAAGGAGCTTTCGAATCTTATGAAGGAGAAGGCGCCGATAAAATA---
TTTCGCCGCTCCCCGACTTGCATTCCATCTGGTCAACCATGCCCC---TACAACGAAAATTGCTGCAGCAAATCG---TGTACATATAAGGAAAATGAAAACGGCAACACTGTTCAAAGATGC------GAC------
>omega-hexatoxin-Hvn1b_3
ATGAATACCGCTACAGGTGTCATCGCTCTTTTGGTTCTGGCGACAGTCATCGGATGCATTGAAGTAGAAGAAACCAGAGCAGATCTTCAAGGAGCTTTCGAATCTTATGAAGGAGAAGCCGCCGAGAAAATA---
TTTCGCCGCTCCCCGACTTGCATTCCATCTGGTCAACCATGCCCC---TACAACGAAAATTGCTGCAGCAAATCG---TGTACATATAAGGAAAATGAAAACGGCAACACTGTTCAAAGATGC------GAC------
>omega-hexatoxin-Hvn1b_4
ATGAATACCGCTACAGGTGTCATCGCTCTTTTGGTTCTGGCGACAGTCATCGGATGCATTGAAGCAGAAGAAACCAGAGCAGATCTTCAAGGAGCTTTCGAATCTTATGAAGGAGAAGCCGCCGATAAAATA---
TTTCGCCGCTCCCCGACTTGCATTCCATCTGGTCAACCATGCCCC---TACAACGAAAATTGCTGCAGCAAATCG---TGTACATATAAGGAAAATGAAAACGGCAACACTGTTCAAAGATGC------GAC------
>omega-hexatoxin-Hvn1b_5
ATGAATACTGCTACAGGTGTCATCGCTCTTTTGGTTCTGGCGACAGTCATCGGATGCATTGAAGCAGAAGAAACCAGAGCAGATCTTCAAGGAGCTTTCGAATCTTATGAAGGAGAAGCCGCCGATAAAATA---
TTTCGCCGCTCCCCGACTTGCATTCCATCTGGTCAACCATGCCCC---TACAACGAAAATTGCTGCAGCAAATCG---TGTACATATAAGGAAAATGAAAACGGCAACACTGTTCAAAGATGC------GAC------
>omega-hexatoxin-Hvn1b_6
ATGAATACTGCTACAGGTGTCATCGCTCTTTTGGTTCTGGCGACAGTCATCGGATGCATTGAAGCAGAAGATACCAGAGCAGATCTTCAAGGAGGTTTGGAATCTTATGAAGGAGAAGCCGCCGATAAAATA---
TTCCGCCGCTCCCCGACTTGCATTCCATCTGGTCAACCATGCCCC---TACAACGAAAATTGCTGCAGCAAATCG---TGTACATATAAGGAAAATGAAAACGGCAACACTGTTCAAAGATGC------GAC------
>omega/kappa-hexatoxin-Ar1g3_2
ATGAATACCGCAACAGGTTTCATCGTACTTTTGGTTTTGGCGACAGTTCTCGGAGGTATTGAAGCT------------------------GGA------GAATCTCATATGAGAAAAGATGCCATGGGAAGA---
GTTCGTCGACAATAT---TGCGTTCCAGTTGATCAACCGTGCTCTCTGAATACCCAACCGTGCTGCGATGATGCCACGTGCACACAAGAGCTAAATGAAAACGACAACACTGTTTATTATTGCAGGGCT---------
>omega/kappa-hexatoxin-Ar1g_1
ATGAATACCGCAACAGGTTTCATCGTCCTTTTGGTTTTGGCGACAGTTCTCGGAGGCATTGAAGCA------------GGAGAATCTCATATGAGAAAAGATGCC---ATGGGAAGAGTT------------------
CGTCGACAATAT---TGCGTTCCAGTTGATCAACCGTGCTCTCTGAATACCCAACCGTGCTGCGATGATGCCACGTGCACACAAGAACTAAATGAAAACGACAACACTGTTTATTATTGCAGGGCT---------

>omega/kappa-hexatoxin-Ar1g_2
ATGAATACCGCAACAGGTTTCATCGTCCTTTTGGTTTTGGCGACAGTTCTCGGAGGTATTGAAGCT------------AGAGAATCTCATATGAGAAAAGATGCC---ATGGGAAGAGTT------------------
CGTCGACAATAT---TGCGTTCCAGTTGATCAACCGTGCTCTCTGAATACCCAACCGTGCTGCGATGATGCCACGTGCACACAAGAGCTAAATGAAAACGACAACACTGTTTATTATTGCAGGGCT---------
>omega/kappa-hexatoxin-Ar1g_3
ATGAATACCGCAACAGGTTTCATCGTACTTTTGGTTTTGGCGACAGTTCTCGGAGGTATTGAAGCT------------GGAGAATCTCATATGAGAAAAGATGCC---ATGGGAAGAGTT------------------
CGTCGACAATAT---TGCGTTCCAGTTGATCAACCGTGCTCTCTGAATACCCAACCGTGCTGCGATGATGCCACGTGCACACAAGAGCTAAATGAAAACGACAACACTGTTTATTATTGCAGGGCT---------
>omega/kappa-hexatoxin-Ar1g_4
ATGAATACCGCAACAGGTTTCATCGTCCTTTTGGTTTTGGCGACAGTTCTCGGAGGTATTGAAGCT------------GGAGAATCTCATATGAGAAAAGATGCC---ATGGGAAGAGTT------------------
CGTCGACAATAT---TGCGTTCCAGTTGATCAACCGTGCTCTCTGAATACCCAACCGTGCTGCGATGATGCCACGTGCACACAAGAGCTAAATGAAAACGACAACACTGTTTATTATTGCAGGGCT---------
>omega/kappa-hexatoxin-Ar1g_5
ATGAATACCGCAACAGGTTTCATCGTCCTTTTGGTTTTGGCGACAGTTCTCGGAGGTATTGAAGCT------------GGAGAATCTCATATGAGAAAAGATGCC---ATGGGAAGAGTT------------------
CGTCGACAATAT---TGCGTTCCAGTTGATCAACCGTGCTCTCTGAATACCCAACCGTGCTGCGATGATGCCACGTGCACACAAGAGCTAAATGAAAACGACAACACTGTTTATTATTGCAGGGCT---------
>omega/kappa-hexatoxin-Hv1g_1
ATGAATACCGCAACAGGTTTCATCGTCCTTTTGGTTTTGGCGACAGTTCTCGGAGGTATTGAAGCA------------GGAGAATCTCATATGAGAAAAGATGCC---ATGGGAAGAGTT------------------
CGTCGACAATAT---TGCGTTCCAGTTGATCAACCGTGCTCTCTGAATACCCAACCGTGCTGCGATGATGCCACGTGCACACAAGAACTAAATGAAAACGACAACACTGTTTATTATTGCAGGGCT---------
>omega/kappa-hexatoxin-Hv1g_2
ATGAATACCGCAACAGGTTTCATCGTCCTTTTGGTTTTGGCGACAGTTCTCGGAGGCATTGAAGCA------------GGAGAATCTCATATGAGAAAAGATGCC---ATGGGAAGAGTT------------------
CGTCGACAATAT---TGCGTTCCAGTTGATCAACCGTGCTCTCTGAATACCCAACCGTGCTGCGATGATGCCACGTGCACACAAGAACTAAATGAAAACGACAACACTGTTTATTATTGCAGGGCT---------
>omega/kappa-hexatoxin-Hv1h
ATGAATACCGCAACAGGTTTCATCGTCCTTTTGGTTTTGGCGACAATTCTCGGAGGTATTGAAGCA------------GGAGAATCTCATATGAGAAAAGATGCC---ATGGGAAGAGTT------------------
CGTCGACAATAT---TGCGTTCCAGTTGATCAACCGTGCTCTCTGAATACCCAACCGTGCTGCGATGATGCCACGTGCACACAAGAGCGGAATGAAAACGGCCACACTGTTTATTATTGCAGGGCT---------
>omega-kappa-hexatoxin-Hv1g7
ATGAATACCGCAACAGGTTTCATCGTCCTTTTGGTTTTGGCGACAGTTCTCGGAGGTATTGAAGCT------------GGAGAATCTCATATGAGAAAAGATGCC---ATGGGAAGAGTT------------------
CGTCGACAATAT---TGCGTTCCAGTTGATCAACCGTGCTCTCTGAATACCCAACCGTGCTGCGATGATGCCACGTGCACACAAGAGCTAAATGAAAACGACAACACTGTTTATTAATGCAGGGTT---------
>omega/kappa-hexatoxin-Hv1g_3
ATGAATACCGCAACAGGTTTCATCGTCCTTTTGGTTTTGGCGACAGTTCTCGGAGGTATTGAAGCA------------GGAGAATCTCATATGAGAAAAGATGCC---ATGGGAAGAGTT------------------
CGTCGACAATAT---TGCGTTCCAGTTGATCAACCGTGCTCTCTGAATACCCAACCGTGCTGCGATGATGCCACGTGCACACAAGAACTAAATGAAAACGACAACACTGTTTATTATTGCAGGGCT---------
>omega/kappa-hexatoxin-Hv1g_4
ATGAATACCGCAACAGGTTTCATCGTCCTTTTGGTTTTGGCGACAGTTCTCGGAGGCATTGAAGCA------------GGAGAATCTCATATGAGAAAAGATGCC---ATGGGAAGAGTT------------------
CGTCGACAATAT---TGCGTTCCAGTTGATCAACCGTGCTCTCTGAATACCCAACCGTGCTGCGATGATGCCACGTGCACACAAGAGCTAAATGAAAACGACAACACTGTTTATTATTGCAGGGCT---------
>omega/kappa-hexatoxin-Hv1g_5
ATGAATACCACAACAGGTTTCATCGTCCTTTTGGTTTTGGCGACAATTCTCGGAGGTATTGAAGCA------------GGAGAATCTCATATGAGAAAAGATGCC---ATGGGAAGAGTT------------------
CGTCGACAATAT---TGCGTTCCAGTTGATCAACCGTGCTCTCTGAATACCCAACCGTGCTGCGATGATGCCACGTGCACACAAGAGCTAAATGAAAACGACAACACTGTTTATTATTGCAGGGCT---------
>omega/kappa-hexatoxin-Hv1g_6
ATGAATACCGCAACAGGTTTCATCGTCTTTTTGGTTTTGGCGACAGTTCTCGGAGGTATTGAAGCA------------GGAGAATCTCATATGAGAAAAGATGCC---ATGGGAAGAGTT------------------
CGTCGACAATAT---TGCGTTCCAGTTGATCAACCGTGCTCTCTGAATACCCAACCGTGCTGCGATGATGCCACGTGCACACAAGAACTAAATGAAAACGACAACACTGTTTATTATTGCAGGGCT---------
>omega-hexatoxin-Hi1g_1
ATGAATACCGCTACAGGTTTCATCGTACTTTTGGTTTTGGCGACAGTGATCGGATGCATTTCTGCTGATTTTGAAGGAAGTTTCGAACCTTATGAAGAAGAAGACGCCGAA---AGAATA---------------
TTTCGCCGCTCAACT---TGCACTCCAACTGATCAACCGTGCCCC---TACGACGAAAGTTGCTGCAGCGGTTCC---TGTACATATAAGGCAAATGAAAACGGCAACCAAGTTAAAAGATGC------GAC------
>omega-hexatoxin-Hi1g_2
ATGAATACCGCTACAGGTTTCATCGTACTTTTGGTTTTGGCGACAGTGATCGGATGTATTTCTGCAGATTTTCAAGGAGGTTTTGAACCTTATGAAGAAGAAGACGCCGAA---AGAATA---------------
TTTCGCCGCTCAACT---TGCACTCCAACTGATCAACCGTGCCCC---TACGACGAAAGTTGCTGCAGCGGTTCC---TGTACATATAAGGCAAATGAAAACGGCAACCAAGTTAAAAGATGC------GAC------
>omega-hexatoxin-Hi1g_3
ATGAATACCGCTACAGGTTTCATCGTTCTTTTGGTTTTGGCGACAGTGATCGGATGCATTTCTGCAGATTTTCAAGGAAGTTTCGAACCTTATGAAGAAGAAGACGCCGAA---AGAATA---------------
TTTCGCCGCTCAACT---TGCACTCCAACTGATCAACCGTGCCCC---TACGACGAAAGTTGCTGCAGCGGTTCC---TGTACATATAAGGCAAATGAAAACGGCAACCAAGTTAAAAGATGT------GAC------
>omega-hexatoxin-Hi1g_4
ATGAATACCGCTACAGGTTTCATCGTACTTTTGGTTTTGGCGACAGTGATCGGATGCATTTCTGCAGATTTTCAAGGAAGTTTCGAACCTTATGAAGAAGAAGACGCCGAA---AGAATA---------------
TTTCGCCGCTCAACT---TGCACTCCAACTGATCAACCGTGCCCC---TACGACGAAAGTTGCTGCAGCGGTTCC---TGTACATATAAGGCAAATGAAAACGGCAACCAAGTTAAAAGATGC------GAC------
>kappa-hexatoxin-Hv1c_2
ATGAATACTGCTACATGTTTCATCGTTCTTTTGGATGTGGCGACTGTCATCGGAGGCATTGAAGCA------------GGAGAATCTGATATGAGAAAAGATGTC---ATGGATTA-------------------
TTTCGCCGAGCTATT---TGCACTGGAGCCGACAGACCGTGCGCGGCGTGCTGC---CCGTGCTGCCCAGGACCTCGTGCAAAGCAGAATCAAAC------GGT------GTTTCTTATTGCAGGAAAGACGAACCT

```
>kappa-hexatoxin-Hv1c_3
ATGAATACTGCTACATGTTTCATCGTTCTTTTGGTTGTGGCGACTGTCATCGGAGGCATTGAAGCA------------GGAGAATTTGATATGAGAAAAGATGTC---ATGGGATTA------------------
TTTCGCCGAGCTATT---TGCACTGGAGCCGACAGACCGTGCGCGGCGTGCTGC---CCGTGCTGCCCAGGGACCTCGTGCAAAGCAGAATCAAAC------GGT------GTTTCTTATTGCAGGAAAGACGAACCT
>kappa-hexatoxin-Hv1c_4
ATGAATACTGCTACATGTTTCATCGTTTTTTTGGTTGTGGCGACTGTCATCGGAGGCATTGAAGCA------------GGAGAATCTGATATGAGAAAAGATGTC---ATGGGATTA------------------
TTTCGCCGAGCTATT---TGCACTGGAGCCGACAGACCGTGCGCGGCGTGCTGC---CCGTGCTGCCCAGGGACCTCGTGCAAAGCAGAATCAAAC------GGT------GTTTCTTATTGCAGGAAAGACGAACCT
>kappa-hexatoxin-Hv1e
ATGAATACTGCTACATGTTTCATCGTTCTTTTGGTTGTGGCGACTGTCATCGGAGGCATTGAAGCA------------GGAGAATTTGATATGAGAAAAGATGTC---ATGGGATTA------------------
TTTCGCCGAGCTATT---TGCCCTGGAGCCGACAGACCGTGCGCGGCGTGCTGC---CCGTGCTGCCCAGGGACCTCGTGCAAAGCAGAATCAAAC------GGT------GTTTTTTATTGCAGGAAAGACGAACCT
>kappa-hexatoxin-Hv1d
ATGAATACTGCTACATGTTTCATCGTTCTTTTGGTTGTGGCGACTGTCATCGGAGGCATTGAAGCA------------GGAGAATCTGATATGAGAAAAGATGTC---ATGGGATTA------------------
TTTCGCCGAGTTATT---TGCACTGGAGCCGACAGCCCGTGCGCGGCGTGCTGC---CCGTGCTGCCCAGGGACCTCGTGCAAAGCAGAATCAAAC------GGT------GTTTCTTATTGCAGGAAAGACGAACCT
>omega-hexatoxin-Hvn1a_2
ATGAATACCGCTACAGGTGTCATCGCTCTTTTGGTTCTGGCGACAGTCATCGGATGCATTGAAGCAGAA---ACCAGAGCAGATCTTCAAGGAGCTTTCGAATCTTATGAAGGAGAAGCCGCCGAGAAAAT---
ATTTCGCCGCTCCCCGACTTGCATTCCATCTGGTCAACCATGCCCC---TACAACGAAAATTGCTGCAGCAAATC---GTGTACATATAAGGAAATGAAAACGGCAACACCTGTTCAAAGATGC------GAC------
>kappa-hexatoxin-Hmo1a
ATGAATACTGCTACAGGTTTCATCGTTCTTTTGGTGTTRGCGACAGTCATCGGAGGCATTGAAGCA------------------------GGA------GAATCTGATATGAGAAAAGACGTCATGGGATTA---TTTCGCCGA---
ACTATTTGCACTGGAGCCGACAGACCGTGCGCAGCGTGCTGC---CCGTGCTGCCCAGGGACCTCGTGC------CAAGGGCCAGAATCAAACWGTATTTCTTAT---TGCAGGAACGAC------
```
